# NF1 and SPRED1/2 cooperate through RAS-MAPK-independent functions

**DOI:** 10.1073/pnas.2535319123

**Published:** 2026-05-27

**Authors:** Jillian M. Silva, Lizzeth Canche, Alice Cheng, Lucy C. Young, Frank McCormick

**Affiliations:** ^a^ Helen Diller Family Comprehensive Cancer Center, University of California San Francisco, San Francisco, CA 94153

**Keywords:** neurofibromin, SPRED, RAS, MAPK

## Abstract

Neurofibromin, the protein product of the neurofibromatosis type 1 (*NF1*) gene, requires the direct binding interaction with SPRED to negatively regulate the RAS-MAPK pathway. Although the region of neurofibromin that stimulates the intrinsic GTPase activity of RAS represents only a small percentage of the entire protein, a large degree of the *NF1* structural domains and their correlating mechanistic functions remain elusive. Here, we demonstrate RAS-independent biochemical and signaling functions regulated by the coordinate control of NF1 and SPRED1/2. Utilizing CRISPR-Cas9 methods to ablate *NF1* or *SPRED1/2* in isogenic “RASless” mouse embryonic fibroblast (MEF) cell lines expressing either the KRAS4b wild-type variant or an oncogenic *KRAS*-mutation, we show loss of SPRED1/2 phenocopies NF1 loss and their cooperation is required to modulate MAPK-AKT signaling. Moreover, NF1 or SPRED1/2 loss also resulted in a potent suppression of the RAS family GTPases, RRAS and RRAS2, occurring independently of RAS or AKT pathway activation. A transcriptome microarray analysis of the NF1 or SPRED1/2 knockout MEF cells revealed a specific subset of RAS-independent, NF1–SPRED1/2-dependent gene signatures, in which these same genes were also directly regulated by the RAS-GTPase function of neurofibromin. The modulation of these NF1–SPRED1/2-dependent downstream signaling effectors were further corroborated in Schwann cell models derived from Neurofibromatosis type I patients that consisted of either plexiform neurofibroma cells or unaffected nerve cells abrogated of NF1 or neurofibromin RAS-GAP activity. Taken together, this study provides RAS-independent functions that are dependent on the cooperation of NF1 and SPRED1/2 in a manner that is uncoupled from canonical MAPK signaling.

## Significance

The RAS-GTPase activating protein (RAS-GAP) related domain of neurofibromin constitutes a small portion of the protein, suggesting that neurofibromin may possess additional functions beyond its well-characterized role in RAS regulation. In this study, we identified several epithelial–mesenchymal transition (EMT)-associated gene signatures that are dependent on the cooperation of neurofibromin and sprouty-related EVH1 domain-containing protein (SPRED) yet function independently of enhanced guanosine triphosphate (GTP)-loading on RAS or sustained activation of MAPK signaling. Notably, we demonstrate that these neurofibromin/SPRED downstream effectors are directly regulated by the RAS-GAP activity of neurofibromin rather than unidentified functions of the protein. These findings reveal a critical role for the neurofibromin/SPRED complex in modulating noncanonical signaling outputs in a manner that is RAS-GAP-dependent but MAPK-independent.

Neurofibromin, encoded by the neurofibromatosis type 1 (*NF1*) gene, is a large dimeric protein that negatively regulates RAS proteins (1–3). It contains a central GTPase-activating protein (GAP) related domain (GRD) that stimulates the GTPase activity of RAS to promote its conversion to the inactive GDP-bound state (4–6). In addition to serving as a GAP for the classical RAS isoforms, Kirsten rat sarcoma viral oncogene homolog (KRAS), Neuroblastoma Ras viral oncogene homolog (NRAS), and Harvey rat sarcoma viral oncogene homolog (HRAS), neurofibromin augments the GTPase activity of the closely related RAS subfamily members, Ras-related protein R-Ras (RRAS), Ras-related protein R-Ras2 (RRAS2), and Muscle Ras oncogene homolog (MRAS) (7). However, neurofibromin requires the binding interaction of another negative regulator of RAS-mitogen-activated protein kinase (MAPK) signaling, SPRED (Sprouty-related EVH1 domain-containing protein), to its GRD flanking sequences to localize it to the plasma membrane, where it abrogates RAS function (8, 9).

Loss of NF1 or SPRED are the underlying causes of two RASopathy syndromes that are characterized by the constitutive activation of the RAS-MAPK signaling pathway. Neurofibromatosis type I (NF1), an autosomal dominant disorder, is caused by inherited or de novo germline mutations in the *NF1* gene (10, 11). There is a wide spectrum of clinical features of NF1 disease that include neurofibromas, café-au-lait macules, axillary freckling, and social, behavioral, and developmental issues (12, 13). Similarly, heterozygous inactivating mutations located in the *SPRED1* gene results in Legius syndrome, an autosomal dominant condition that shares similar phenotypic features with NF1 but lacks the more severe clinical manifestations (8). Overlapping functions of the two additional SPRED isoforms (SPRED2 and SPRED3) has been hypothesized to compensate for the loss of SPRED1 as these isoforms can also bind neurofibromin to regulate RAS-MAPK activity and may account for Legius syndrome resembling a more moderate version of NF1 disease (14, 15).

While the region of neurofibromin that regulates RAS activity represents only a small fraction of the entire protein, a large extent of the neurofibromin functional domains remain uncharacterized. The widespread distribution of mutations in NF1 disease encompasses regions throughout neurofibromin that are unrelated to its RAS-GAP domain, suggesting that neurofibromin possesses RAS-independent functions and whose disruption may substantially contribute to the pathogenesis of the disease. Here, we explored the potential regulatory functions of neurofibromin beyond its canonical role in modulating RAS by employing CRISPR-Cas9 to abrogate *NF1* or *SPRED1/2* in isogenic mouse embryonic fibroblast (MEF) cells lacking the primary RAS isoforms (KRAS, NRAS, and HRAS) and expressing either the wild-type variant or an activating oncogenic mutation in KRAS4b. Direct comparison of wild-type and oncogenic *KRAS*-mutant MEFs in NF1- or SPRED1/2-deficient cells enabled the differentiation between the RAS-dependent vs. the RAS-independent functions mediated by the NF1–SPRED1/2 complex. We observed that loss of SPRED1/2 phenocopies NF1 loss resulting in sustained RAS-MAPK activity. Interestingly, loss of NF1 or SPRED1/2 also led to a robust suppression of the RAS-GTPase subfamily members, RRAS and RRAS2, that occurred independent of MAPK or AKT pathway activation. A transcriptome microarray analysis revealed a substantial downregulation of gene signatures that are independent of canonical RAS signaling yet dependent on NF1 and SPRED1/2, in which the RAS-GAP function of neurofibromin is critical in the regulation of these transcriptional outputs. Thus, these data support the rationale that NF1 and SPRED1/2 cooperate to regulate a cohort of RAS-independent signaling effectors exclusive of MAPK pathway activation.

## Results

### Loss of NF1 or SPRED1/2 Leads to Elevated RAS Activity.

**Fig. 1.**
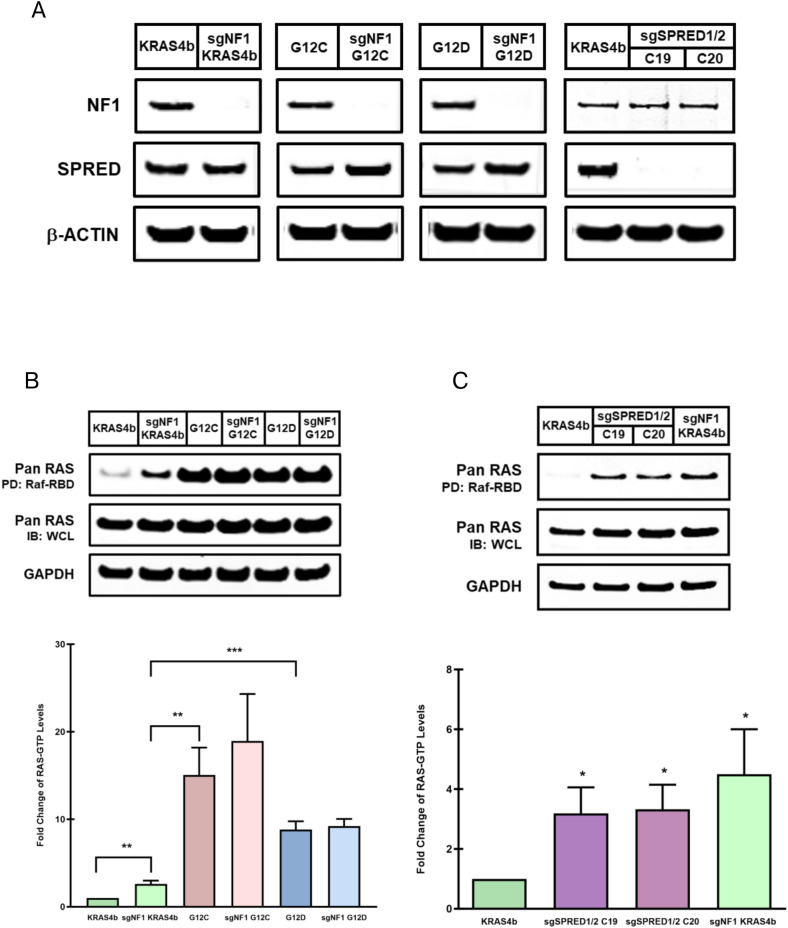
The effect of NF1 or SPRED1/2 loss on RAS-GTP levels. (*A*) Lysates from the sgNF1 and sgSPRED1/2 (clones 19 and 20) knockout MEF cells were analyzed by immunoblotting with the indicated antibodies to confirm deletion of NF1 or SPRED protein levels. (*B* and *C*) RAS-GTP levels were measured by pulling down the GTP-bound RAS/Raf-RBD complexes from the sgNF1 (*B*) or sgSPRED1/2 (*C*) knockout MEF lysates followed by immunoblotting with pan RAS (above). Quantification of RAS-GTP levels are presented as mean ± SEM of three or more independent experiments (below). *P* values were determined by unpaired, two-tailed *t* tests (*P < 0.05; **P < 0.01; ***P < 0.001). PD: pull-down, IB: immunoblot, WCL: whole cell lysate.

The key function of neurofibromin is to stimulate the conversion of the active GTP-bound form of RAS to its inactive GDP-bound state (4–6, 16). Hence, the loss of neurofibromin results in increased RAS-GTP levels (17–20). Therefore, to directly assess whether NF1 or SPRED loss further enhances RAS-GTP levels, we ablated NF1 or SPRED1/2 in isogenic RASless MEF cells reconstituted with a single wild-type KRAS4b allele or an oncogenic KRAS^G12C^ or KRAS^G12D^ mutant allele and lacking both HRAS and NRAS proteins. This cell model provides a genetically defined system with minimal genetic background variations without the input or compensatory actions from the other two major RAS proteins (21). To dissect the RAS-dependent from the RAS-independent effects on the signaling and biochemical functions of NF1 and SPRED1/2, we abrogated NF1 in two different oncogenic *KRAS*-mutant MEF cells to exclude the possibility that NF1 may phenocopy an activating KRAS mutation. Deletion of NF1 or SPRED1/2 in the KRAS4b^WT^ (KRAS4b hereafter), KRAS^G12C^, and KRAS^G12D^ MEF cell lines were verified by NGS and Western blot analyses (Fig. 1*A* and *SI Appendix*, Tables S2 and S3).

RAS-GTP levels were analyzed by pulling down GTP-bound RAS/Raf-RBD complexes from sgNF1 knockout KRAS4b, KRAS^G12C^, and KRAS^G12D^ MEF cells. Loss of NF1 in the KRAS4b MEF cells led to a significant increase in RAS-GTP levels compared to their parental cells without affecting their proliferation, in which this increase in RAS activation was further corroborated in three additional independent sgNF1 knockout KRAS4b MEF clones (Fig. 1*B* and *SI Appendix*, Fig. S1 *A* and *B*). NF1 loss in the KRAS^G12C^ or KRAS^G12D^ MEF cells did not result in a significant elevation in RAS-GTP levels or proliferation compared to their respective parental cell line (Fig. 1*B* and *SI Appendix*, Fig. S1*B*). However, the KRAS^G12C^ and KRAS^G12D^ MEF cells displayed a substantial greater activation of RAS and proliferative rate compared to the sgNF1 knockout KRAS4b MEF cells. SPRED1/2 loss in the KRAS4b MEF cells also exhibited a significant elevation in RAS-GTP levels compared to their parental cells, however, both the RAS-GTP levels and proliferative rate remained markedly lower than those observed in the sgNF1 knockout KRAS4b MEF cells (Fig. 1*C* and *SI Appendix*, Fig. S1*C*). Thus, consistent with prior reports, genetic ablation of NF1 or SPRED1/2 leads to sustained accumulation of RAS-GTP, underscoring the critical role of the NF1–SPRED1/2 complex in regulating aberrant RAS activation.

### NF1 or SPRED1/2 Loss Is Required for Sustained MAPK and AKT Activation in the Absence of the RAS-Related GTPases, RRAS and RRAS2.

**Fig. 2.**
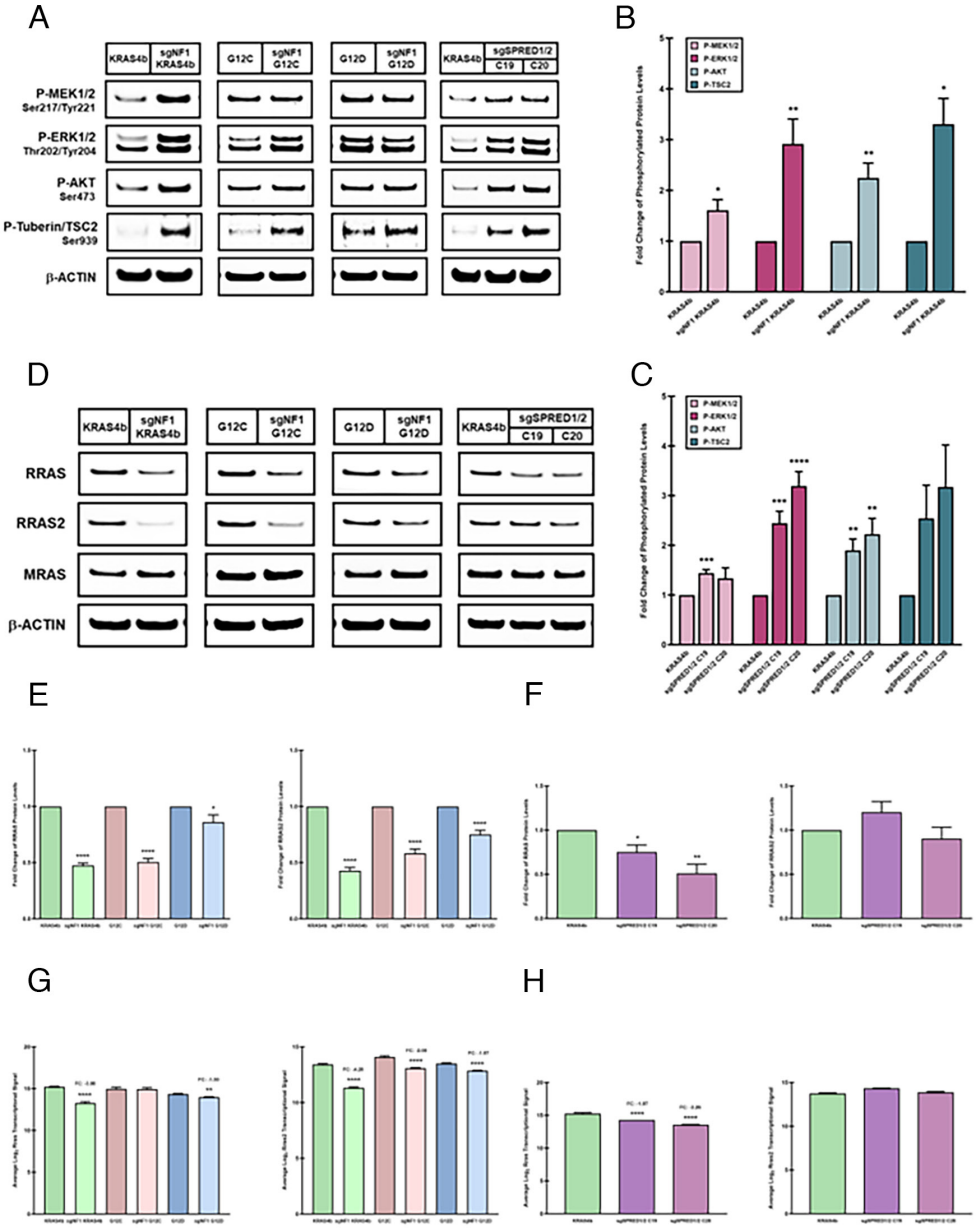
Consequences of NF1 or SPRED1/2 deletion on signaling downstream of RAS. (*A*) sgNF1 and sgSPRED1/2 (clones 19 and 20) knockout MEF lysates were analyzed by immunoblotting with the indicated antibodies. (*B* and *C*) Quantification of the phosphorylated proteins examined in (*A*) from the sgNF1 (*B*) or sgSPRED1/2 (*C*) knockout KRAS4b MEF cells with data presented as mean ± SEM of three or more independent experiments. Unpaired, two-tailed *t* tests were used to determine *P* values (*P < 0.05; **P < 0.01; ***P < 0.001; ****P < 0.0001). (*D*) sgNF1 and sgSPRED1/2 knockout MEF lysates were analyzed by immunoblotting with the RRAS, RRAS2, or MRAS antibodies. (*E* and *F*) Quantification of the RRAS and RRAS2 protein levels from the sgNF1 (*E*) or sgSPRED1/2 (*F*) knockout MEF cells analyzed in (*D*) with data presented as mean ± SEM of five or more independent experiments. Unpaired, two-tailed *t* tests were used to determine *P* values (*P < 0.05; **P < 0.01; ****P < 0.0001). (*G* and *H*) Rras and Rras2 transcript levels in the sgNF1 (*G*) or sgSPRED1/2 (*H*) knockout MEF cells are presented as an average Log_2_ signal and analyzed using a SST-RMA algorithm with *P* values determined by one-way ANOVA analyses (**P < 0.01; ****P < 0.0001). SST-RMA: single-space transformation-robust multichip analysis, FC: fold change.

Since it has also been demonstrated that loss of NF1 leads to the prolonged activation of MAPK signaling, we examined the consequences of NF1 or SPRED1/2 loss on signaling downstream of RAS in the sgNF1 and sgSPRED1/2 knockout MEF cells (22, 23). As expected, loss of NF1 or SPRED1/2 led to a profound increase in mitogen-activated protein kinase kinase 1 and 2 (MEK1/2) phosphorylation and over a twofold elevation in extracellular signal-regulated kinases 1 and 2 (ERK1/2) phosphorylation in both the sgNF1 and sgSPRED1/2 knockout KRAS4b MEF cells compared to their parental cells (Fig. 2 *A*–*C*). This increase in MEK1/2 and ERK1/2 phosphorylation was further verified in the additional independent sgNF1 knockout KRAS4b MEF clones (*SI Appendix*, Fig. S2 *A* and *B*). The sgNF1 knockout KRAS^G12C^ MEF cells led to a moderate increase in ERK1/2 phosphorylation (1.5-fold) without any discernible effect on MEK1/2 phosphorylation compared to the parental KRAS^G12C^ cells (Fig. 2*A*). However, loss of NF1 in the KRAS^G12D^ MEF cells had no effect on MEK1/2 or ERK1/2 phosphorylation levels compared to its parental cells (Fig. 2*A*). Interestingly, sgNF1 or sgSPRED1/2 knockout KRAS4b MEF cells both exhibited over a twofold increase in AKT phosphorylation and its direct downstream target, tuberin/tuberous sclerosis complex 2 (TSC2) (Fig. 2 *A*–*C* and *SI Appendix*, Fig. S2 *A* and *B*). Although loss of NF1 in the KRAS^G12C^ and KRAS^G12D^ MEF cells did not result in a further increase in AKT phosphorylation, NF1 loss in both activating *KRAS*-mutant cell lines displayed a modest increase in tuberin/TSC2 phosphorylation compared to their corresponding parental cell line (Fig. 2*A*). Moreover, it has also been demonstrated that NF1 loss can activate the mammalian target of rapamycin complex 1 (mTORC1) pathway through the inactivation of the hamartin/tuberous sclerosis complex 1 (TSC1)/TSC2 complex via AKT-mediated phosphorylation (24–28). Despite the elevation in AKT and tuberin/TSC2 phosphorylation in the sgNF1 and sgSPRED1/2 knockout KRAS4b MEF cells, there was no detectable effect on any of the downstream mTORC1 effectors, 70-kDa ribosomal S6 kinase (p70^S6K)^, ribosomal protein S6 (S6RP), or eukaryotic translation initiation factor 4E-binding protein 1 (4E-BP1) (*SI Appendix*, Fig. S3*A*). Hence, these data suggest that the loss of SPRED1/2 phenocopies NF1 loss and the cooperation of NF1 and SPRED1/2 is necessary to regulate MAPK and AKT signaling.

Previous studies have shown that PI3K-AKT signaling is a major downstream effector pathway regulated by RRAS and RRAS2 (29–32). Moreover, *Nf1*-deficient mouse Schwann cell precursors exhibited increased levels of AKT phosphorylation and additional loss of RRAS2 in these cells correlated with a reduction in AKT phosphorylation (33). Therefore, we analyzed whether the elevation in AKT phosphorylation is a result of RRAS/RRAS2 modulation. Surprisingly, all sgNF1 knockout MEF cells, regardless of the presence of an activating *KRAS* mutation, displayed a profound decrease in RRAS and RRAS2 at both the transcript and protein levels compared to their respective parental cell line with little or no effect on MRAS levels (Fig. 2 *D*, *E*, and *G* and *SI Appendix*, Fig. S2). Similarly, sgNF1 knockout KRAS4b MEF cells also exhibited a substantial decrease in RRAS- and RRAS2-GTP levels, whereas MRAS-GTP levels were unaffected compared to their parental cells (*SI Appendix*, Fig. S3*B*). SPRED1/2 loss also led to a substantial reduction in RRAS transcript and protein levels but exhibited negligible effects on RRAS2 or MRAS levels (Fig. 2 *D*, *F*, and *H*). Furthermore, these data indicate that NF1 and SPRED1/2 cooperate to regulate RRAS and RRAS2 signaling independent of AKT activation.

### Ablation of NF1 or SPRED1/2 Downregulates a Subset of Gene Signatures Independent of RAS Activity.

**Fig. 3.**
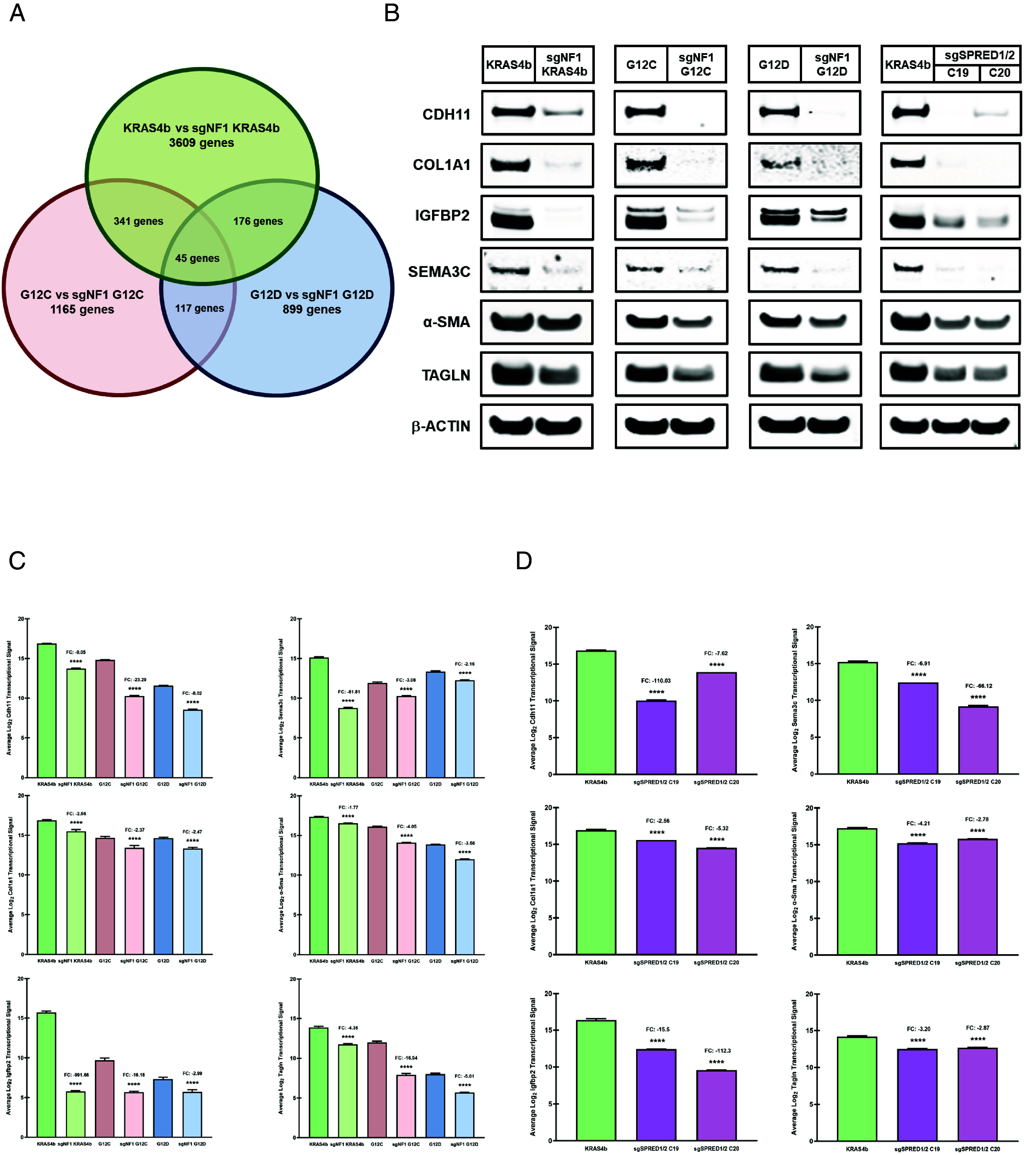
NF1–SPRED1/2-dependent, RAS-independent gene signatures. (*A*) Venn diagram of the number of common genes from the Clariom™ D transcriptome microarray analysis between each sgNF1 knockout MEF cell line and their respective parental cell line and a direct comparison among all three sgNF1 knockout MEF datasets. Transcriptome data analysis required a twofold increase or decrease in gene expression and a *P* value of ≤ 0.05. (*B*) NF1–SPRED1/2-dependent signaling was analyzed in the sgNF1 and sgSPRED1/2 (clones 19 and 20) knockout MEF cell lysates by immunoblotting with the indicated antibodies. (*C* and *D*) Transcript levels of the genes examined in (*B*) from the sgNF1 (*C*) or sgSPRED1/2 (*D*) knockout MEF cells are presented as an average Log_2_ signal and analyzed using a SST-RMA algorithm with *P* values determined by one-way ANOVA analyses (****P < 0.0001). SST-RMA: single-space transformation-robust multichip analysis, FC: fold change.

To further investigate the genes that may contribute to the RAS-independent signaling mechanisms of NF1 and SPRED1/2, we performed a Clariom™ D transcriptome microarray analysis with the sgNF1 and sgSPRED1/2 knockout MEF cell lines. This analysis provides the most intricate gene and exon level expression profiles including the ability to detect rare and low-expressing transcripts by utilizing the most comprehensive and largest number of transcriptome databases. Analysis criteria of transcriptome data required a twofold change (≤−2 or ≥2) in gene expression with a statistically significant *P* value of less than 0.05. Transcript data from each sgNF1 knockout MEF cell line were compared with their respective parental cell line and then each of these datasets was compared among each other to elucidate the NF1-dependent, RAS-independent genes (Fig. 3*A*). The transcriptome analysis identified 45 genes common among the sgNF1 knockout MEF cells, which was comprised of 28 downregulated and 17 upregulated genes (*SI Appendix*, Table S1). Of the 45 NF1-dependent genes, the sgSPRED1/2 knockout KRAS4b MEF clones 19 and 20 shared 24 and 30 genes in common, respectively. The transcriptome profiling revealed a specific subset of these NF1–SPRED1/2-dependent genes are associated with the epithelial–mesenchymal transition (EMT). In contrast to previous reports that demonstrate the upregulation of these EMT-related gene signatures are essential for promoting tumorigenesis in several types of cancer, loss of NF1 or SPRED1/2 led to a downregulation of these same genes (34–41). To corroborate the transcriptome data, we performed immunoblotting analyses in the sgNF1 and sgSPRED1/2 knockout MEF cells to examine the protein levels of the following EMT-associated genes, cadherin 11 (CDH11), collagen type 1 alpha 1 (COL1A1), insulin-like growth factor binding protein 2 (IGFBP2), semaphorin 3c (SEMA3C), alpha smooth muscle actin (α-SMA), and transgelin (TAGLN), which were selected solely based on their NF1–SPRED1/2 transcriptional dependency (Fig. 3 *B*–*D* and *SI Appendix*, Fig. S4). Loss of NF1 or SPRED1/2 led to a 75% or greater reduction in CDH11 and COL1A1 protein levels consistent with the profound decrease in transcript levels. IGFBP2 protein levels were substantially inhibited by 60% or more in the sgNF1 and sgSPRED1/2 knockout MEF cells, which complemented the potent suppression of Igfbp2 transcript levels in these cells. NF1 loss displayed a robust decrease in SEMA3C protein levels, but loss of SPRED1/2 resulted in over an 80% reduction in SEMA3C protein levels with all sgNF1 and sgSPRED1/2 knockout MEF cells significantly attenuating Sema3C transcript levels. Loss of NF1 or SPRED1/2 also led to robust decreases in both α-SMA and TAGLN protein and transcript levels, but not to the same extent as observed in the other NF1–SPRED1/2-dependent effectors. Moreover, the additional sgNF1 knockout KRAS4b MEF clones further corroborated the potent suppression of CDH11, COL1A1, SEMA3C, α-SMA, and TAGLN at both the transcript and protein levels (*SI Appendix*, Fig. S5). Furthermore, these data suggest that NF1 and SPRED1/2 regulate a subset of gene signatures that are dependent on the NF1–SPRED1/2 complex, but independent of RAS-MAPK activity.

### Loss of NF1 or Disruption of Neurofibromin’s RAS-GAP Function in NF1 Patient-Derived Nerve or Plexiform Neurofibroma Cells Corroborates the Suppression of the NF1–SPRED1/2-Dependent Signaling Effectors.

**Fig. 4.**
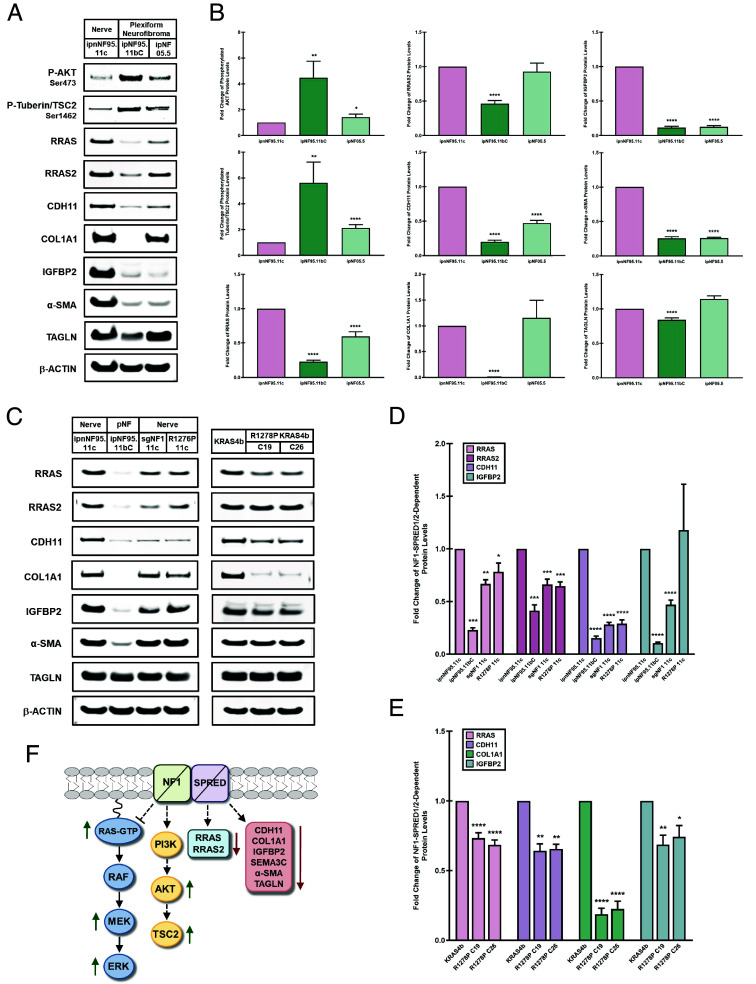
Dependency of the NF1–SPRED1/2 downstream signaling effectors on the RAS-GAP activity of neurofibromin. (*A*) Unaffected nerve (ipnNF95.11c) and plexiform neurofibroma (ipNF95.11bC; ipNF05.5) cell lysates were analyzed by immunoblotting with the indicated antibodies. (*B*) Quantification of the protein levels examined in (*A*) are presented as mean ± SEM of three or more independent experiments. *P* values were determined by unpaired, two-tailed *t* tests (*P < 0.05; **P < 0.01; ****P < 0.0001). (*C*) Lysates from the nerve cells (ipnNF95.11c), plexiform neurofibroma cells (ipNF95.11bC), sgNF1 knockout (sgNF1 11c) or NF1^R1276P^ mutant (R1276P 11c) nerve cells, and NF1^R1278P^ mutant KRAS4b MEF cells (clones 19 and 26) were analyzed by immunoblotting with the indicated antibodies. pNF: plexiform neurofibroma. (*D* and *E*) Quantification of RRAS, RRAS2, CDH11, and IGFBP2 protein levels in nerve cells, plexiform neurofibroma cells, sgNF1 knockout or NF1^R1276P^ mutant nerve cells (*D*), and NF1^R1278P^ mutant KRAS4b MEF cells (*E*) are presented as mean ± SEM of three or more independent experiments. Unpaired, two-tailed *t* tests were employed to determine *P* values (*P < 0.05; **P < 0.01; ***P < 0.001; ****P < 0.0001). (*F*) Schematic illustrating the downstream signaling consequences of NF1 or SPRED1/2 loss.

Since the modulation of the NF1–SPRED1/2-dependent gene signatures were demonstrated to be under the coordinate control of the NF1–SPRED1/2 complex in an isogenic MEF model, we sought to substantiate these findings in NF1^Null^ plexiform neurofibroma cells derived from Neurofibromatosis type I patients. A comparison of Schwann cells from an unaffected peripheral nerve (ipnNF95.11c) and nonmalignant plexiform neurofibroma tumors (ipNF95.11bC and ipNF05.5) revealed that both plexiform neurofibroma cell lines exhibited substantial increases in AKT phosphorylation and its direct downstream targets, PRAS40 and tuberin/TSC2, as previously demonstrated in the sgNF1 and sgSPRED1/2 knockout KRAS4b MEF cells (Fig. 4 *A* and *B* and *SI Appendix*, Fig. S6 *A* and *B*). Interestingly, the elevation in AKT and tuberin/TSC2 phosphorylation correlated with a significant increase in the phosphorylation of the mTORC1 downstream target, 4E-BP1, in the plexiform neurofibroma cell lines with largely no effect on p70^S6K^ or ribosomal protein S6 phosphorylation levels (*SI Appendix*, Fig. S6 *A* and *B*). Analogous to the sgNF1 and sgSPRED1/2 knockout MEF cells, both plexiform neurofibroma cell lines exhibited profound decreases in RRAS, CDH11, IGFBP2, and α-SMA protein levels, but only the ipNF95.11bC plexiform neurofibroma cell line displayed a potent suppression of RRAS2, COL1A1, and TAGLN protein levels (Fig. 4 *A* and *B*). Moreover, these data were also corroborated in NF1^Null^ melanoma cells, which exhibited significant decreases in RRAS, RRAS2, CDH11, and IGFBP2 protein levels with modest effects on α-SMA and TAGLN protein levels (*SI Appendix*, Fig. S6 *C* and *D*). Hence, these data emphasize the ability of NF1 to regulate these noncanonical RAS outputs in a manner that is independent of AKT activation.

To assess whether the RAS-GTPase function of neurofibromin regulates the NF1–SPRED1/2-dependent signature genes, we introduced a CRISPR-engineered missense mutation (NF1^R1278P^) into the arginine finger of the GAP-related domain (GRD) of neurofibromin in the KRAS4b MEF cells. This specific arginine finger mutant does not impair neurofibromin’s protein structure, cellular levels, or ability to effectively bind RAS, but selectively and completely abolishes its RAS-GAP function (42, 43). The NF1^R1278P^ mutant KRAS4b MEF cells exhibited robust decreases in RRAS, CDH11, COL1A1, and IGFBP2 protein levels as previously observed in the sgNF1 and sgSPRED1/2 knockout MEF cells (Fig. 4 *C* and *E*). In contrast, the NF1^R1278P^ mutant KRAS4b MEF cells only displayed modest reductions in α-SMA and TAGLN protein levels with no effect on RRAS2 levels (Fig. 4*C* and *SI Appendix*, Fig. S7*B*).

The dependency of neurofibromin’s GAP activity on the NF1–SPRED1/2 downstream signaling effectors was further evaluated by engineering both a CRISPR-knockin of the arginine finger mutation (NF1^R1276P^) and a CRISPR-Cas9 sgNF1 knockout in unaffected nerve cells derived from a NF1 patient (ipnNF95.11c). The sgNF1 knockout or NF1^R1276P^ mutant nerve cells exhibited profound decreases in RRAS, RRAS2, and CDH11 protein levels, consistent with the reduction of these proteins in the plexiform neurofibromin cell lines (Fig. 4 *C* and *D*). The sgNF1 knockout nerve cells also potently suppressed IGFBP2 protein levels as demonstrated in both plexiform neurofibroma cell lines, whereas the NF1^R1276P^ mutant nerve cells did not modulate IGFBP2 levels (Fig. 4 *C* and *D*). In contrast to the plexiform neurofibroma cells, the sgNF1 knockout and NF1^R1276P^ mutant nerve cells displayed negligible effects on COL1A1, α-SMA, and TAGLN protein levels (Fig. 4*C* and *SI Appendix*, Fig. S7*A*). In comparison to NF1^R1278P^ mutant KRAS4b MEF cells, the NF1^R1276P^ mutant nerve cells only shared similar reductions in RRAS and CDH11 protein levels, which may be attributed to the mutational composition of the nerve cells compared to the isogenic MEF cells. Furthermore, these data indicate that the NF1–SPRED1/2-dependent downstream gene signatures are, at least in part, modulated by the RAS-GTPase function of neurofibromin, which is unrelated to its ability to suppress RAS activity.

## Discussion

Loss of neurofibromin or its binding partner SPRED results in the sustained activation of the canonical RAS proteins, consequently leading to the dysregulation of the MAPK signaling pathway. However, it remains elusive whether noncanonical RAS signaling pathways may also be impacted by the loss of NF1 or SPRED and contribute to the pathogenesis associated with NF1 disease or Legius syndrome. Moreover, the elucidation of these RAS-independent mechanisms may be obscured by the indirect consequences of RAS activation or the compensatory functions mediated by the other primary RAS isoforms. To address this issue, we identified alterations in the cellular signaling profiles in which RAS pathway activation is unaffected by the loss of neurofibromin.

NF1 loss in isogenic MEF cells only expressing the oncogenic KRAS^G12C^ or KRAS^G12D^ mutants did not further alter RAS-GTP levels or MAPK signaling compared to their respective parental cell lines rendering these *KRAS*-mutant cells resistant to neurofibromin GTPase activity. Consistent with previous reports, RAS mutations prevent the arginine finger of GAPs from entering the GTPase site in RAS to promote GTP hydrolysis and thereby resulting in increased GTP-loading and MAPK activation (44, 45). Conversely, loss of NF1 or SPRED1/2 in KRAS4b^WT^ MEF cells exhibited elevated RAS-GTP levels and enhanced MAPK pathway activation, but the magnitude of RAS activation was comparatively lower than that observed in the oncogenic *KRAS*-mutant MEF cells (Fig. 4*F*).

Although loss of NF1 or SPRED1/2 resulted in AKT activation and the deregulation of the TSC1/TSC2 complex, we did not observe any corresponding changes in the downstream mTORC1 effectors (Fig. 4*F*). Despite the established role of NF1 in regulating mTORC1 signaling, these data suggest that the coordinate control of AKT modulation by both NF1 and SPRED1/2 may occur through noncanonical AKT-mTORC1 mechanisms (27, 28). Since AKT contributes to an extensive and diverse repertoire of cellular signaling pathways and functions, over a hundred AKT substrates may serve as potential candidates of the NF1–SPRED1/2-mediated regulation of AKT (46, 47). Hence, parsing out which of these downstream effector pathways is important for the cooperation of NF1 and SPRED1/2 requires further exploration.

To identify the non-RAS-mediated functions of neurofibromin, a comprehensive transcriptome analysis of NF1 or SPRED1/2 loss in wild-type KRAS4b or *KRAS*-mutant MEF cells identified a cohort of RAS-independent gene expression signatures regulated by the cooperation of NF1 and SPRED1/2. Although a subset of these gene signatures, CDH11, COL1A1, IGFBP2, SEMA3C, α-SMA, and TAGLN, are associated with EMT and tumor promotion, their potent suppression upon loss of NF1 or SPRED1/2 suggests that these genes may perform alternative, context-dependent functions that are modulated by the NF1–SPRED1/2 axis (Fig. 4*F*) (34–41). Accordingly, the downregulation of these EMT-related genes likely represents a direct cell-intrinsic transcriptional consequence of NF1 loss, rather than a reflection of intact neurofibromas comprised of heterogenous cell populations of Schwann cells, fibroblasts, endothelial, immune, and mast cells all of which collectively contribute to the tumor microenvironment (48, 49). Furthermore, loss of expression of this cohort of NF1–SPRED1/2-dependent genes was also observed across three distinct NF1^Null^ cellular models, including NF1 patient-derived plexiform neurofibroma cells, sgNF1 knockout peripheral nerve cells generated from a NF1 patient, and NF1^Null^ melanoma cell lines. These findings provide compelling evidence that NF1 and SPRED1/2 cooperate to regulate a shared transcriptional output in addition to modulating RAS-MAPK activity.

Moreover, we also demonstrate that the NF1–SPRED1/2 complex plays a critical role in the regulation of the RAS-GTPase subfamily members, RRAS and RRAS2, in which loss of NF1 or SPRED1/2 inhibited both GTPases independent of RAS or AKT activation (Fig. 4*F*). Accordingly, abrogation of the RAS-GAP function of neurofibromin not only displayed an analogous effect of suppressing the NF1–SPRED1/2-dependent downstream effectors but also downregulated RRAS and RRAS2 as observed in the sgNF1 and sgSPRED1/2 knockout MEF and nerve cells. These biochemical and signaling effects indicate that these NF1–SPRED1/2-dependent signature outputs are mediated by the RAS-GAP activity of neurofibromin rather than attributed to the loss of an entirely separate function of the protein. Furthermore, knockdown of the p120 RAS-GAPs, Rasa1, or Rasa2, which also retain the ability to suppress RAS function, showed no discernible impact on any of the NF1–SPRED1/2 downstream effectors further supporting the hypothesis that modulation of these gene signatures is exclusively governed by neurofibromin (*SI Appendix*, Fig. S8).

These data collectively illustrate that NF1 and SPRED1/2 cooperate to regulate a distinct set of EMT-related gene signatures that are independent of the enhanced GTP-loading on the canonical RAS proteins and activation of the MAPK pathway. We propose that neurofibromin may serve as a GAP for an undisclosed GTPase, whose function regulates the expression of this distinct cohort of genes that may be involved in EMT or alternative signaling mechanisms that remain to be characterized. The extent to which these additional non-RAS functions contribute to the NF1 phenotype will be the subject of future investigative studies. In conclusion, this study provides the identification of RAS-independent, NF1-dependent proteins that may serve as potential biomarkers and molecular targets for the development of prospective therapeutic agents for the treatment of NF1 and Legius syndrome patients.

## Material and Methods

### Cell Culture.

Isogenic “RASless” MEF cell lines expressing a single wild-type KRAS4b or an oncogenic *KRAS*-mutant allele were obtained from the well-curated cell line repository established by the Frederick National Laboratory of the National Cancer Institute (Frederick, MD) and maintained in DMEM, high glucose media supplemented with 10% FBS, penicillin/streptomycin, and 4 µg/mL blasticidin (*SI Appendix*, Table S2) (21). Authentication of the MEF cell lines was performed by the Frederick National Laboratory using whole-exome sequencing, PCR, and immunoblotting methods. Unaffected peripheral nerve-derived (hTERT NF1 ipnNF95.11c; CRL-3391™) and plexiform neurofibroma-derived (hTERT NF1 ipNF95.11b C; CRL-3390™ and hTERT NF1 ipNF05.5; CRL-3388™) Schwann cells from NF1 patients were purchased from ATCC and cultured in DMEM, high glucose media containing 10% FBS and penicillin/streptomycin with authentication and characterization performed as previously described (*SI Appendix*, Table S2) (50). All cell lines were subjected to *Mycoplasma* testing using the MycoAlert™ *Mycoplasma* detection kit (Lonza) on a routine basis.

### Generation of the NF1 and SPRED1/2 CRISPR-Cas9 Knockout Cells.

The CRISPR-Cas9 NF1 knockout cell lines were generated in isogenic MEF cells reconstituted with the KRAS4b^WT^ or oncogenic KRAS^G12C^ or KRAS^G12D^ mutant allele or NF1 patient-derived unaffected nerve cells, ipnNF95.11c, whereas the CRISPR-Cas9 sgSPRED1/2 knockout cells were generated in the isogenic KRAS4b^WT^ MEF cells (*SI Appendix*, Tables S2 and S3). Three CRISPR in vitro transcribed (IVT) sgRNAs were synthesized using the GeneART™ precision gRNA synthesis kit (A29377) for the sgNF1 and sgSPRED1/2 knockout MEF cell lines and the MEGAshortscript™ T7 transcription kit (AM1354) for the sgNF1 knockout ipnNF95.11c nerve cells with all sgRNAs purified using MEGAclear™ transcription clean-up kit (AM1908) according to the manufacturer’s instructions (ThermoFisher Scientific) (*SI Appendix*, Table S4). The MEF knockout cell lines were transfected with each CRISPR IVT gRNA using either GeneART™ Platinum™ Cas9 protein (sgNF1 and sgSPRED1/2 KRAS4b^WT^) or TrueCut™ Cas9 v2 protein (A36496) (sgNF1 KRAS^G12C^ and sgNF1 KRAS^G12D^) and Lipofectamine® CRISPRMAX™ for 72 h. The sgNF1 knockout ipnNF95.11c nerve cells were transfected with each CRISPR IVT gRNA and TrueCut™ Cas9 v2 protein by electroporation with the Neon® transfection system (MPK10025) according to the manufacturer’s protocol (ThermoFisher Scientific). In addition, asymmetrical single-stranded DNA oligo donors with phosphorothioate modifications at the 5’ and 3’ ends were also synthesized to induce an in-frame stop codon in the NF1 gene of the ipnNF95.11c nerve cells and the SPRED1 and SPRED2 genes of the KRAS4b^WT^ MEF cells (ThermoFisher Scientific) (*SI Appendix*, Table S4).

Next-Generation Sequencing (NGS) analysis was performed to determine the on-target cleavage efficiency and potential off-target cleavage events using ThermoFisher Scientific’s proprietary CRISPR Designer Tool, which did not detect any off-target cleavage candidates in any of the sgNF1 or sgSPRED1/2 knockout stable pools (*SI Appendix*, Table S5). Single-cell cloning of the sgNF1 or sgSPRED1/2 knockout stable pools with the highest editing efficiency was performed by limited dilution procedure at a density of 0.5, 1, 2, and 3 cell(s) per well in 96-well plates. NGS and Western blot analyses verified complete knockout of NF1 or SPRED1 and SPRED2 in either the sgNF1 or sgSPRED1/2 knockout clones (ThermoFisher Scientific) (Fig. 1*A* and *SI Appendix*, Table S3).

### Generation of the NF1 Arginine Finger GRD Knockin Mutants.

The NF1 arginine finger GRD mutants were generated by knocking in a NF1^R1276P^ (human) or NF1^R1278P^ (mouse) SNP in the ipnNF95.11c nerve cells or KRAS4b^WT^ MEF cells, respectively, using CRISPR-Cas9 editing methods (ThermoFisher Scientific) (*SI Appendix*, Tables S2 and S3). Two IVT gRNAs were synthesized using MEGAshortscript™ T7 transcription kit and purified via MEGAclear™ transcription clean-up kit according to the manufacturer’s recommendations (ThermoFisher Scientific) (*SI Appendix*, Table S4). Asymmetrical single-stranded DNA oligo donors were also synthesized to induce an in-frame stop codon in the NF1 gene of the KRAS4b^WT^ MEF and the ipnNF95.11c nerve cells (*SI Appendix*, Table S4). KRAS4b^WT^ MEF and ipnNF95.11c nerve cells were transfected with their respective CRISPR IVT gRNAs, ss-Oligo donor, and TrueCut™ Cas9 v2 protein by electroporation using the Neon® transfection system for 72 h according to the manufacturer’s protocol (ThermoFisher Scientific). NGS analysis was performed to determine the on-target editing efficiency and potential off-target cleavage events using ThermoFisher Scientific’s proprietary in-house CRISPR Design Tool, in which no off-target cleavage was detected (*SI Appendix*, Table S5). Single-cell cloning of the stable pools with the highest editing efficiency was performed via limited dilution procedure at a density of 0.5, 1, 2, and 3 cell(s) per well in 96-well plates and confirmation of the homozygous human NF1^R1276P^ or mouse NF1^R1278P^ mutational edits were verified by NGS analysis (ThermoFisher Scientific) (*SI Appendix*, Table S3).

### Immunoblot Analysis.

Cell lysates were prepared for immunoblot analysis as previously described (51, 52). Thirty micrograms of protein were separated using NuPAGE™ 4-12% Bis-Tris or 3 - 8% Tris-Acetate mini protein gels (ThermoFisher Scientific) and transferred to PVDF membrane using the iBlot™2 dry blotting system (ThermoFisher Scientific). Membranes were blocked in Intercept® PBS blocking buffer (LI-COR Biosciences) and probed with the primary antibodies as described (*SI Appendix*, Table S6). Antigen–antibody complexes were detected using fluorescent goat anti-rabbit or anti-mouse IRDye® 800CW or goat anti-rabbit or anti-mouse IRDye® 680LT IgG secondary antibodies (LI-COR Biosciences) and visualized using the LI-COR Odyssey Classic infrared imaging system. Immunoblot data was analyzed using the Odyssey application software v3.0.30 (LI-COR Biosciences) (52).

### RAS-GTP Pull-Down Activation Assay.

The sgNF1 or sgSPRED1/2 knockout MEF cells and their corresponding parental cell lines were seeded at a density of 2.5 × 10^6^ or 3.0 × 10^6^ cells per 10 cm^2^ dish. The following day, fresh medium was added to each plate for an additional 24 h prior to lysis with Cell Lysis Buffer (50 mM Tris pH 7.5, 10 mM MgCl_2_, 0.5 M NaCl, 2% IGEPAL) containing protease and phosphatase inhibitors (Pierce/ThermoFisher Scientific) according to the manufacturer’s protocol (Cytoskeleton). Lysates were clarified by centrifugation at 10,000 rpm for 2 min at 4 °C and then immediately snap frozen in liquid nitrogen. An aliquot of each lysate was used to measure protein concentrations with the BCA protein assay kit (Pierce/ThermoFisher Scientific). For the RAS activation assays, 300 μg of total cell protein was used to pull-down GTP-bound RAS/Raf-RBD complexes according to the manufacturer’s instructions (Cytoskeleton). Activated RAS or 20 μg of total cell protein was separated using NuPAGE™ 4 - 12% Bis-Tris mini protein gels (ThermoFisher Scientific) and transferred to PVDF membrane using the iBlot™ 2 dry blotting system (ThermoFisher Scientific). Membranes were blocked in Intercept® PBS blocking buffer (LI-COR Biosciences) and probed with the primary antibodies as described (*SI Appendix*, Table S6). Antigen–antibody complexes were detected using fluorescent goat anti-rabbit or anti-mouse IRDye 800CW or goat anti-rabbit or anti-mouse IRDye 680LT IgG secondary antibodies (LI-COR Biosciences) and visualized with the LI-COR Odyssey Classic infrared imaging system. Immunoblot data was analyzed using the Odyssey application software v3.0.30 (LI-COR Biosciences) (52).

### Clariom™ D Transcriptome Microarray Analysis.

Total RNA was extracted in triplicate for each sgNF1 or sgSPRED1/2 knockout MEF cell line and their respective parental cells using the MagMAX™ *mir*Vana™ Total RNA Isolation Kit (A27828) and processed on a KingFisher™ Magnetic Particle Processor (5400110) using the script A27828_FLEX_Tissue_Cells according to the manufacturer’s protocol (ThermoFisher Scientific). RNA concentrations and ratios were measured using the NanoDrop 8000 and RNA integrity was analyzed with the Agilent 6000 RNA Nano kit (ThermoFisher Scientific). ThermoFisher Scientific genome services performed the Clariom™ D mouse transcriptome microarray analyses on all processed RNA samples and conducted a quality assessment of the raw data to ensure all QC metrics performed as expected (*SI Appendix*, Fig. S9). To determine differentially expressed genes, arrays were analyzed using a summarization single space transformation-robust multichip analysis (SST-RMA) algorithm and the Transcriptome Analysis Console (TAC) software 4.0.1 (ThermoFisher Scientific).

### Statistical Analysis.

All quantitative data are represented as mean ± SEM of at least three or more independent experiments. GraphPad Prism 9.4.1 statistical software was used to determine *P* values by performing paired, two-tailed *t* tests or one-way ANOVA analyses as indicated.

## Supplementary Material

Appendix 01 (PDF)

## Data Availability

Study data are included in the article and/or *SI Appendix*.
