# Supplementary material for: NF1 and SPRED1/2 cooperate through RAS-MAPK-independent functions
Source: Proc Natl Acad Sci U S A. 2026 May 27;123(22):e2535319123. doi: 10.1073/pnas.2535319123 (PMC13229218; doi:10.1073/pnas.2535319123)
Supplement: Supplementary file 1 — Appendix 01 (PDF) [file pnas.2535319123.sapp.pdf]

PNAS

Supporting Information for:

NF1 and SPRED1/2 cooperate through RAS-MAPK-independent functions

Jillian M. Silva, Lizzeth Canche, Alice Cheng, Lucy C. Young, and Frank McCormick*

***Correspondence:** Frank McCormick

***Email:** frank.mccormick@ucsf.edu

This PDF file includes:

- SI Materials and Methods
- Figures S1 to S9
- Tables S1 to S6
- SI References

SUPPLEMENTARY MATERIALS AND METHODS

Cell Culture

Human melanoma cell lines were obtained from the following sources: WM3622 and WM3918 from Rockland Immunochemicals, MeWo from ATCC (HTB-65), COLO792 from Millipore-Sigma/ECACC (93052616), and M230, D04, and WM793 cells were kindly provided by Dr. Antoni Ribas (University of California, Los Angeles), Dr. Susana Ortiz-Urada (University of California, San Francisco), and Dr. Meenhard Herlyn (Wistar Institute), respectively (SI Appendix Table S2). WM793, WM3622, and WM3918 cells were maintained in DMEM, high glucose media; MeWo cells were cultured in MEMα medium; and COLO792, M230, and D04 cells were maintained in RPMI 1640 media with all media types supplemented with 10% FBS and penicillin/streptomycin. Authentication and characterization of all human melanoma cells were executed by their respective company or as previously described (1-6). All cell lines were subjected to *Mycoplasma* testing using the MycoAlert™ *Mycoplasma* detection kit (Lonza) on a routine basis.

Cell Proliferation Assay

Short-term cell proliferation was assessed by seeding sgNF1 or sgSPRED1/2 MEF cells at a density of 5.8×10^4 cells in 12-well plates or 1.5×10^5 cells in 6-well plates. Viable cells were fixed and stained using crystal violet at 24, 48 and 72 hours. To quantify cellular proliferation, crystal violet-stained cells were solubilized in 33% acetic acid with absorbance measured at a wavelength of 562 nm.

siRNA Transfection Experiments

$KRAS4b^{WT}$ MEF cells were transfected with three different Rasa1 or Rasa2 *Silencer*®Select siRNAs (Ambion®) and a non-targeting negative control *Silencer*®Select siRNA (ThermoFisher Scientific) at a 10nM concentration using Lipofectamine™ RNAiMAX reagent in Opti-MEM™ reduced serum medium according to the manufacturer's instructions (ThermoFisher Scientific). After 72-hours post-transfection, cells were prepared for immunoblot analysis as previously described (7, 8). Thirty micrograms of protein were separated using NuPAGE™ 4-12% Bis-Tris mini protein gels (ThermoFisher Scientific) and transferred to PVDF membrane using the iBlot™2 dry blotting system (ThermoFisher Scientific). Membranes were blocked in Intercept® PBS blocking buffer (LI-COR

Biosciences) and probed with the primary antibodies as described (SI Appendix Table S6). Antigen-antibody complexes were detected using fluorescent goat anti-rabbit IRDye® 800CW or goat anti-mouse IRDye® 680LT IgG secondary antibodies (LI-COR Biosciences) and visualized using the LI-COR Odyssey Classic infrared imaging system. Immunoblot data was analyzed using the Odyssey application software v3.0.30 software (LI-COR Biosciences) (8).

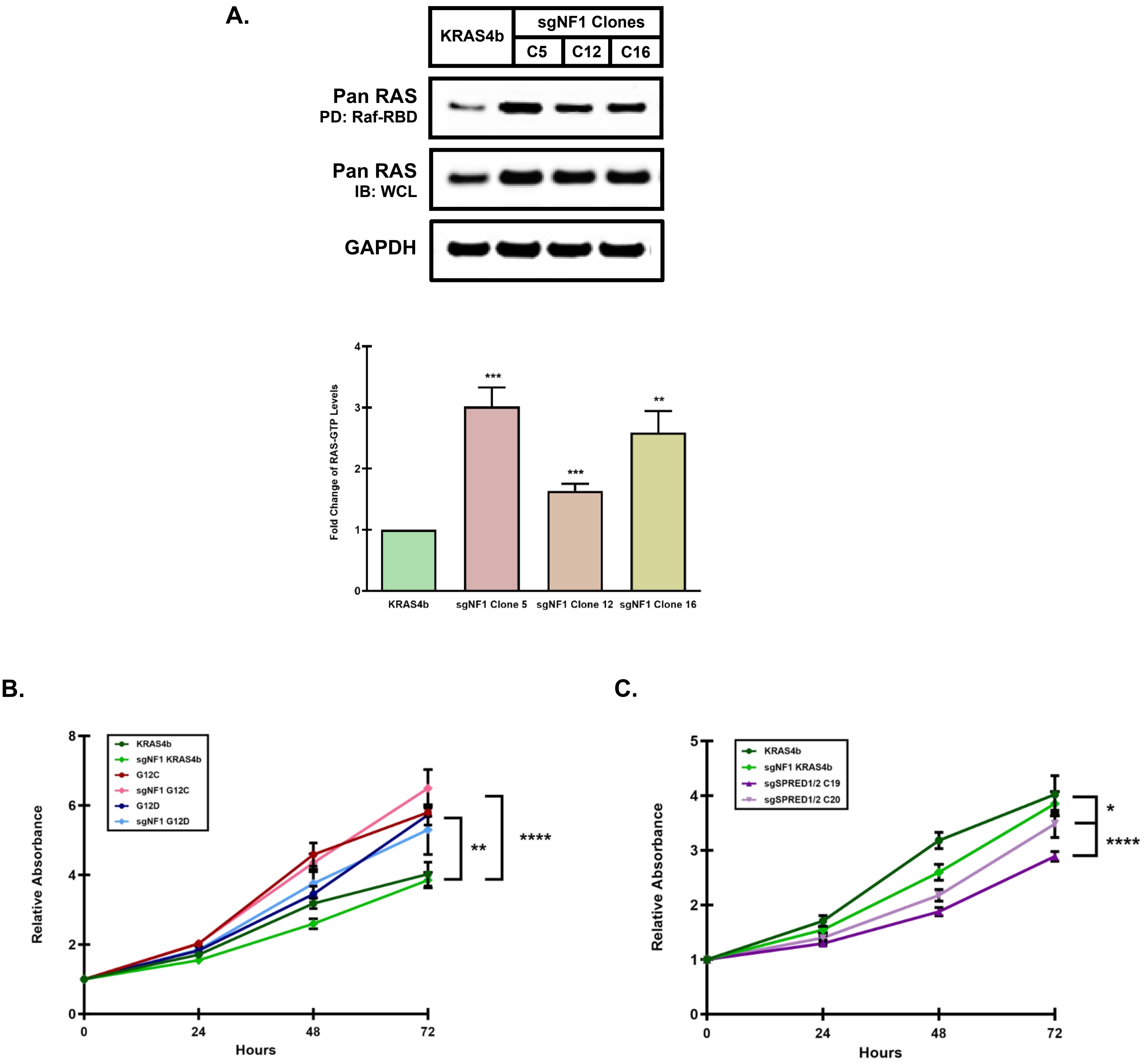

Figure S1. RAS-GTP pull-down assay in the sgNF1 knockout KRAS4b MEF clones and the effect of NF1 or SPRED1/2 loss on MEF proliferation.
(A) RAS-GTP levels were measured by pulling down the GTP-bound RAS/Raf-RBD complexes from the lysates of the sgNF1 knockout KRAS4b MEF clones 5, 12, and 16 followed by immunoblotting with pan RAS (above). Quantification of RAS-GTP levels are presented as mean ± SEM of three or more independent experiments (below). Unpaired, two-tailed *t* tests were used to determine *P* values (***P* < 0.01; ****P* < 0.001). PD: pull-down, IB: immunoblot, WCL: whole cell lysate. **(B)** and **(C)** Short-term proliferation was assessed in the sgNF1 (B) or sgSPRED1/2 (C) knockout MEF cells at 24, 48, and 72 hours with viable cells fixed and stained with crystal violet and quantified by solubilizing the fixed dye and measuring absorbance at λ = 562 nm. Data are presented as mean ± SEM of four or more independent experiments. Two-way ANOVA analyses were performed to determine *P* values (**P* < 0.05; ***P* < 0.01; *****P* < 0.0001).

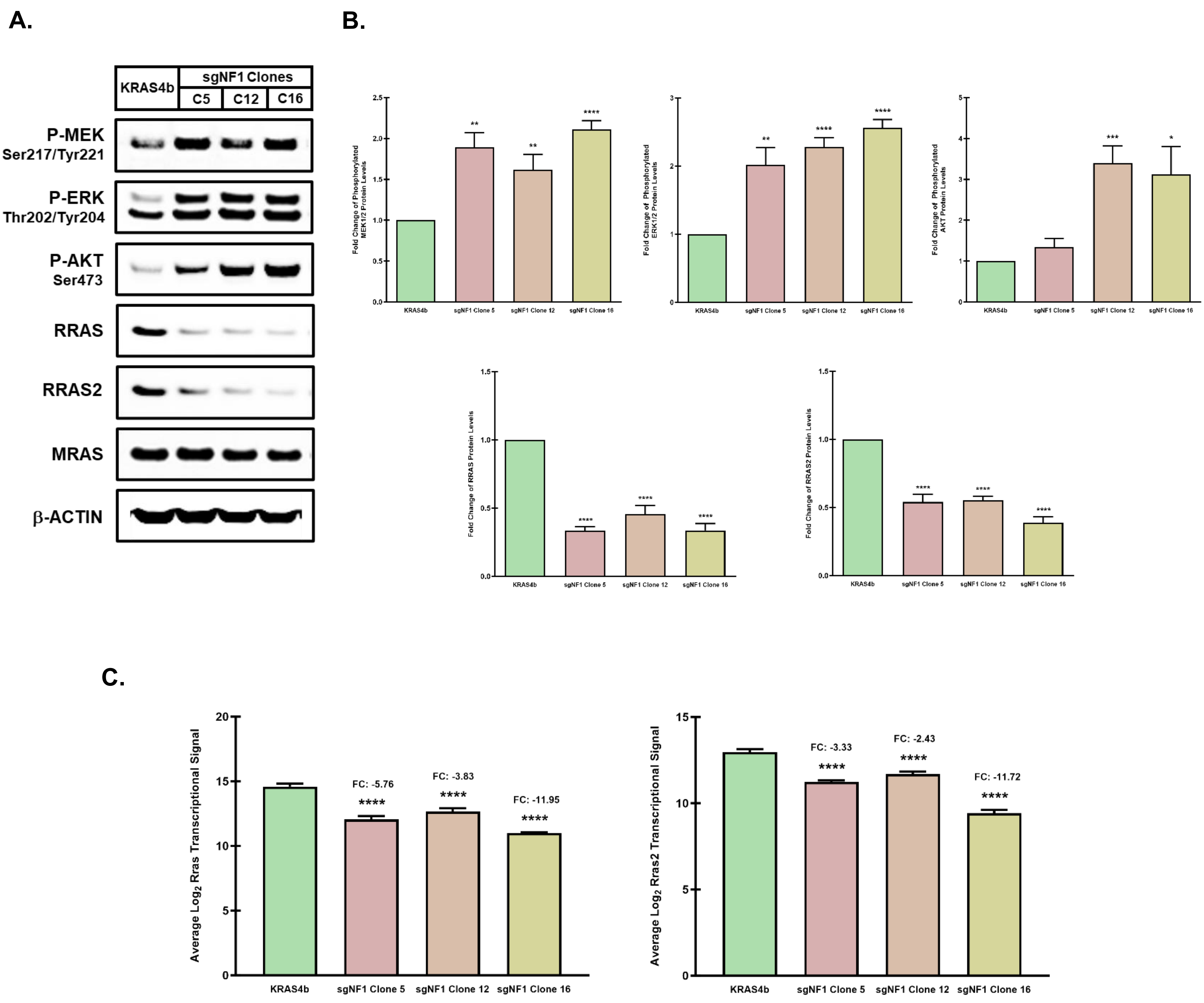

Figure S2. The effect of NF1 loss on protein and transcript levels downstream of RAS. **(A)** Lysates from the sgNF1 knockout KRAS4b MEF clones 5, 12, and 16 were analyzed by immunoblotting with the indicated antibodies. **(B)** Protein quantification of the indicated antibodies analyzed in (A) are presented as mean ± SEM of three or more independent experiments. Unpaired, two-tailed *t* tests were used to determine *P* values (*$P < 0.05$; **$P < 0.01$; ***$P < 0.001$; ****$P < 0.0001$). **(C)** Rras and Rras2 transcript levels in the sgNF1 knockout KRAS4b MEF clones 5, 12, and 16 are presented as an average Log_2 signal and analyzed using a SST-RMA algorithm with *P* values determined by one-way ANOVA analyses (****$P < 0.0001$). SST-RMA: single-space transformation-robust multi-chip analysis, FC: fold change.

A.

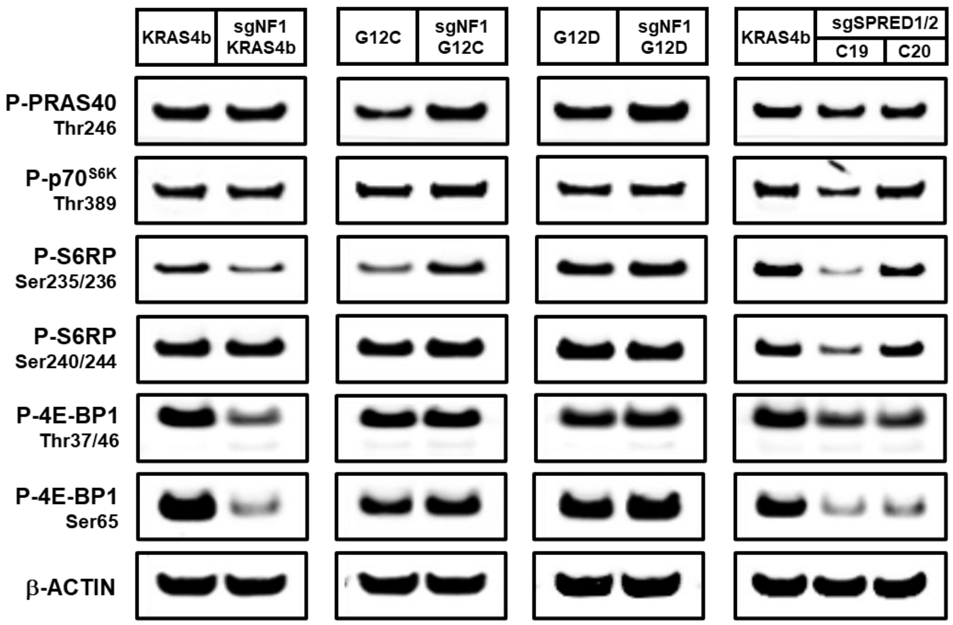

B.

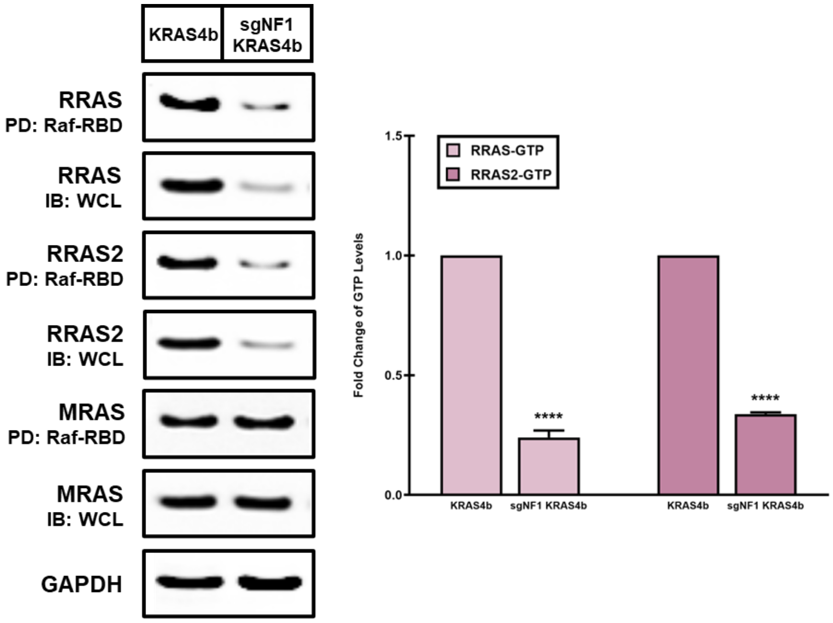

Figure S3. Loss of NF1 or SPRED1/2 on the mTORC1 signaling effectors and GTP levels of the RAS-related GTPases, RRAS, RRAS2, and MRAS.
(A) Lysates from the sgNF1 or sgSPRED1/2 (clones 19 and 20) knockout MEF cells were analyzed by immunoblotting with the indicated antibodies. **(B)** RRAS, RRAS2, and MRAS-GTP levels were measured by pulling down the GTP-bound RRAS, RRAS2, or MRAS/Raf-RBD complexes from the sgNF1 knockout KRAS4b MEF lysates followed by immunoblotting with the indicated antibodies (left). Quantification of RRAS- or RRAS2-GTP levels are presented as mean ± SEM of three independent experiments (right). Unpaired, two-tailed *t* tests were used to determine *P* values (****$P < 0.0001$). PD: pull-down, IB: immunoblot, WCL: whole cell lysate.

A.

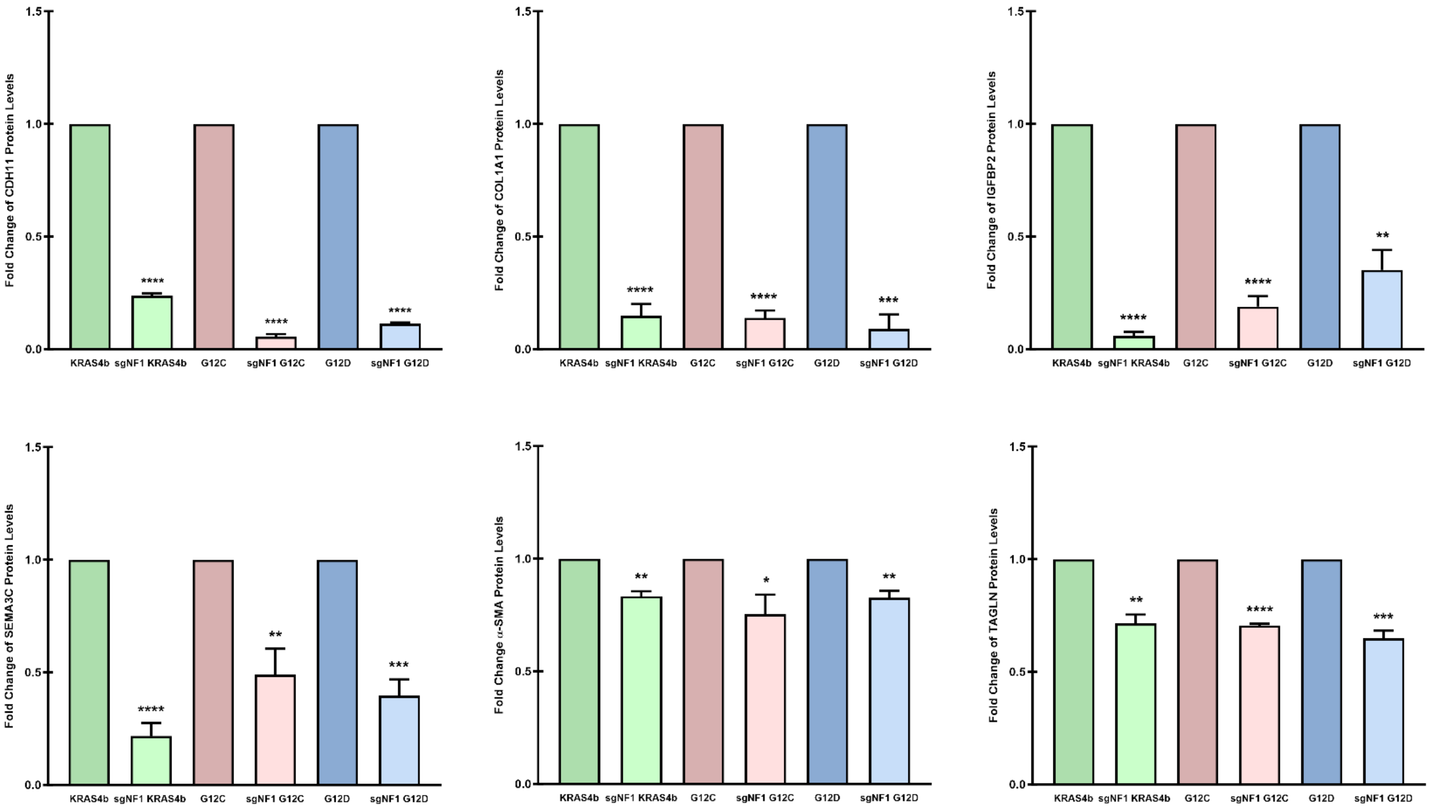

B.

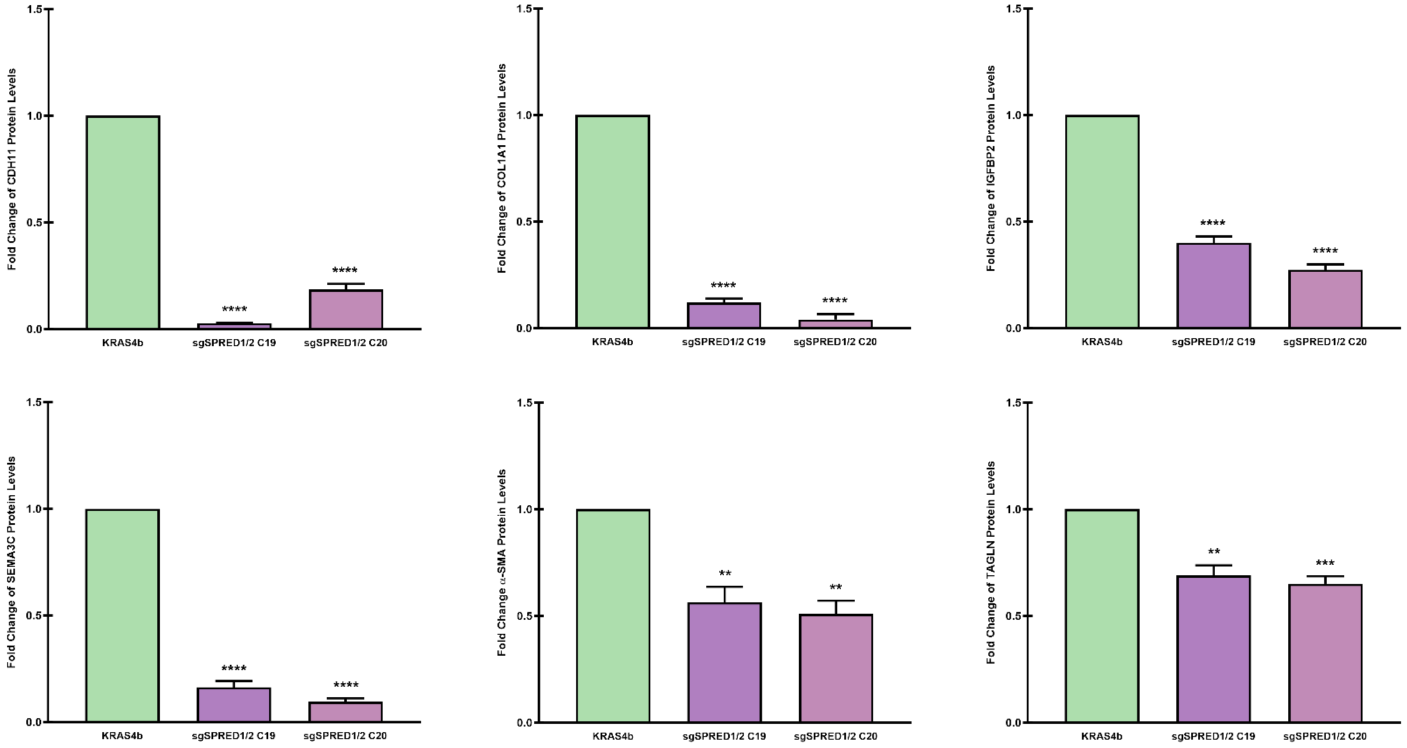

Figure S4. Protein quantification of the NF1-SPRED1/2-dependent gene signatures in the sgNF1 and sgSPRED1/2 knockout MEF cells.
(A) and **(B)** Quantification of CDH11, COL1A1, IGFBP2, SEMA3C, α-SMA, and TAGLN protein levels in the sgNF1 (A) or sgSPRED1/2 (clones 19 and 20) (B) knockout MEF cells are presented as mean ± SEM of three or more independent experiments. Unpaired, two-tailed *t* tests were used to determine *P* values (**P* < 0.05; ***P* < 0.01; ****P* < 0.001; *****P* < 0.0001).

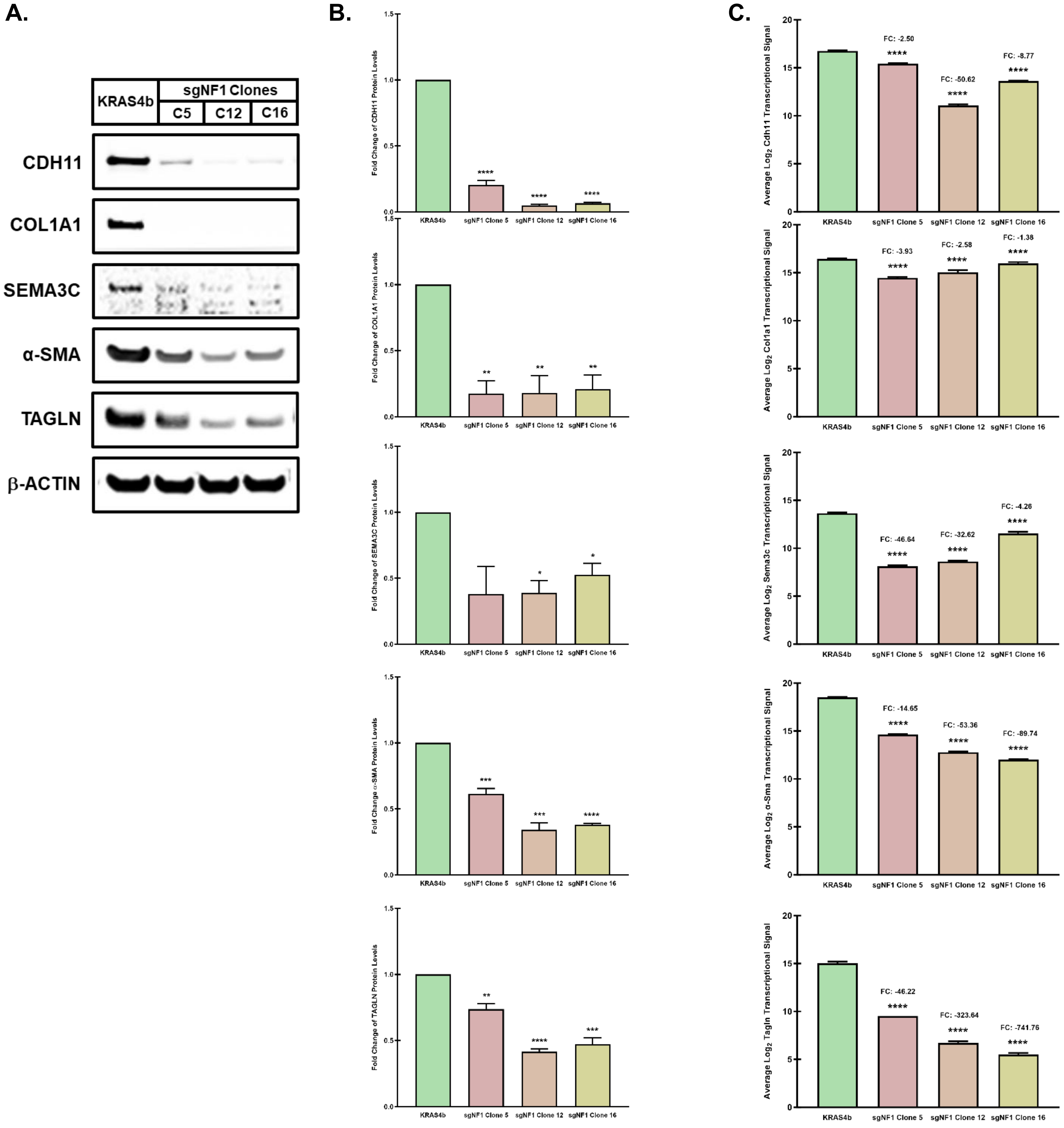

Figure S5. Protein and transcript levels of the NF1-SPRED1/2-dependent gene signatures in the sgNF1 knockout KRAS4b MEF clones.
(A) Lysates from the sgNF1 knockout KRAS4b MEF clones 5, 12, and 16 were analyzed by immunoblotting with the indicated antibodies. **(B)** Quantification of the antibodies analyzed in (A) are presented as mean ± SEM of two or more independent experiments. *P* values were determined by unpaired, two-tailed *t* tests (**P* < 0.05; ***P* < 0.01; ****P* < 0.001; *****P* < 0.0001). **(C)** sgNF1 knockout KRAS4b MEF clones 5, 12, and 16 transcript levels of the antibodies examined in (A) are presented as an average Log_2 signal and analyzed using a SST-RMA algorithm with *P* values determined by one-way ANOVA analyses (*****P* < 0.0001). SST-RMA: single-space transformation-robust multi-chip analysis, FC: fold change.

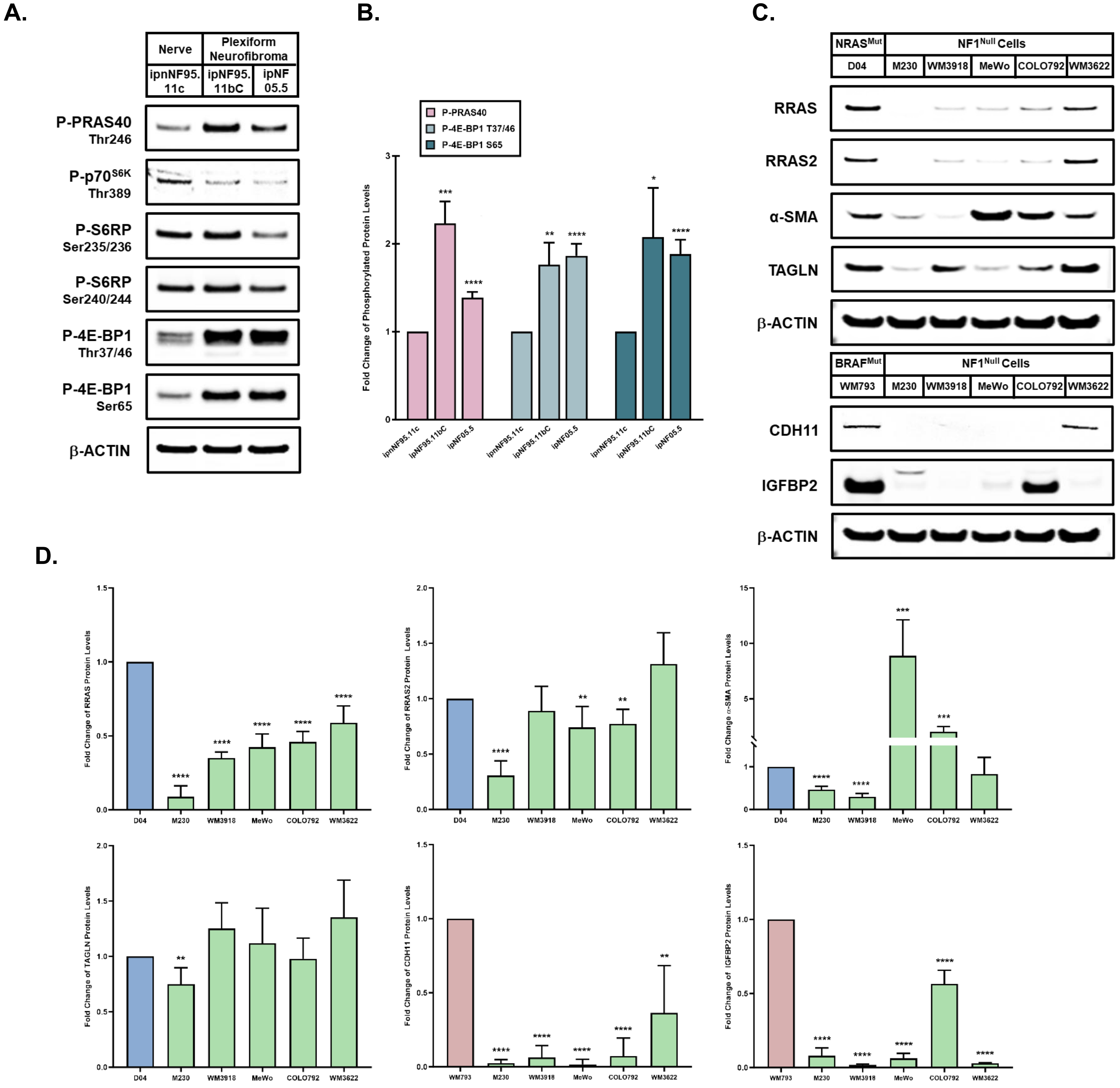

Figure S6. Analysis of AKT-mTORC1 signaling in plexiform neurofibroma cells or NF1-SPRED1/2-dependent signaling effectors in NF1^{Null} melanoma cells.
(A) Lysates from unaffected nerve (ipnNF95.11c) and plexiform neurofibroma tumor (ipNF95.11bC; ipNF05.5) cells were immunoblotted with the indicated antibodies. **(B)** Quantification of phosphorylated PRAS40 and 4E-BP1 protein levels in the nerve and plexiform neurofibroma cells are presented as mean ± SEM of three or more independent experiments. **(C)** Cell lysates from NF1^{Null} (M230, WM3918, MeWo, COLO792, WM3622), NRAS^{Q61L} (D04), or BRAF^{V600E} (WM793) melanoma cell lines were analyzed by immunoblotting with the indicated antibodies. **(D)** Protein quantification of the antibodies analyzed in (C) from the NF1^{Null} melanoma cells are presented as mean ± SEM of three or more independent experiments. All *P* values were determined by unpaired, two-tailed *t* tests (**P* < 0.05; ***P* < 0.01; ****P* < 0.001; *****P* < 0.0001).

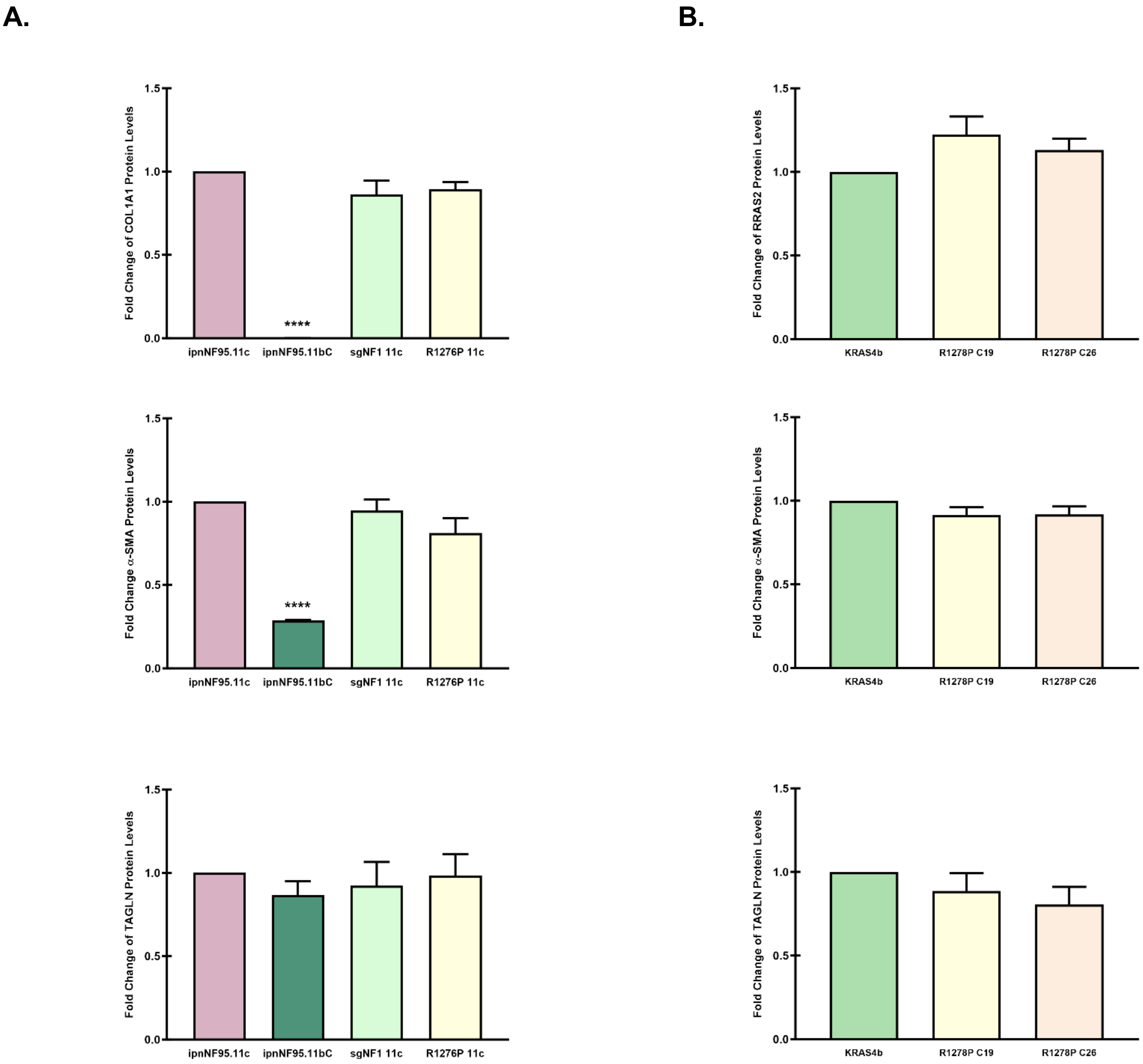

Figure S7. Protein quantification of the NF1-SPRED1/2-dependent gene signatures in the NF1 arginine finger GRD mutant nerve or KRAS4b MEF cells.
(A) Quantification of COL1A1, α-SMA, and TAGLN protein levels in the unaffected nerve cells (ipnNF95.11c), plexiform neurofibroma cells (ipNF95.11bC), and sgNF1 knockout or NF1^{R1276P} mutant nerve cells are presented as mean ± SEM of four independent experiments. *P* values were determined by unpaired, two-tailed *t* tests (*****P* < 0.0001). **(B)** Quantification of RRAS2, α-SMA, and TAGLN protein levels in the NF1^{R1278P} mutant KRAS4b MEF cells (clones 19 and 26) are presented as mean ± SEM of three or more independent experiments.

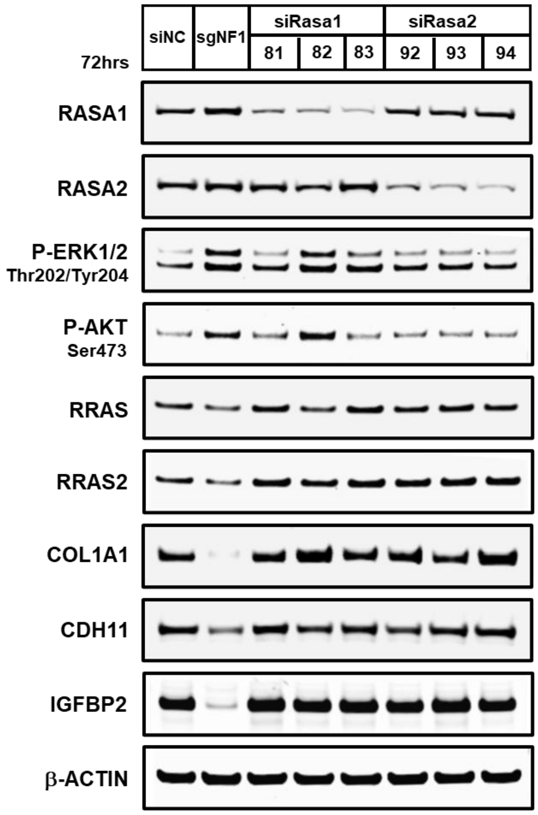

Figure S8. siRNA knockdown of the p120 RAS-GAPs, Rasa1 and Rasa2, on the NF1-SPRED1/2-dependent downstream effectors in KRAS4b MEF cells.
KRAS4b^{WT} MEF cells were treated with either a non-targeting negative control (siNC) or three different siRNAs against Rasa1 (siRasa1) or Rasa2 (siRasa2) for 72 hours and compared to the sgNF1 knockout KRAS4b MEF cells with lysates analyzed by immunoblotting with the indicated antibodies.

A.

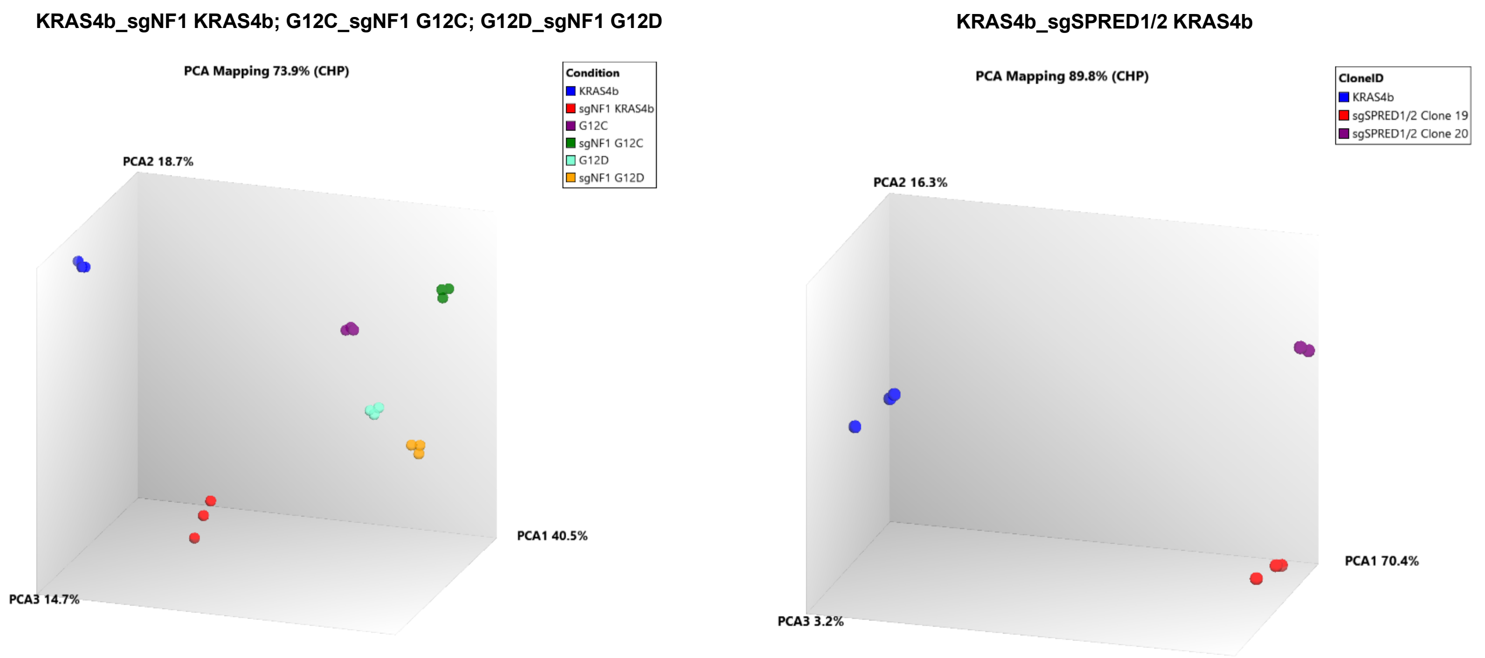

B.

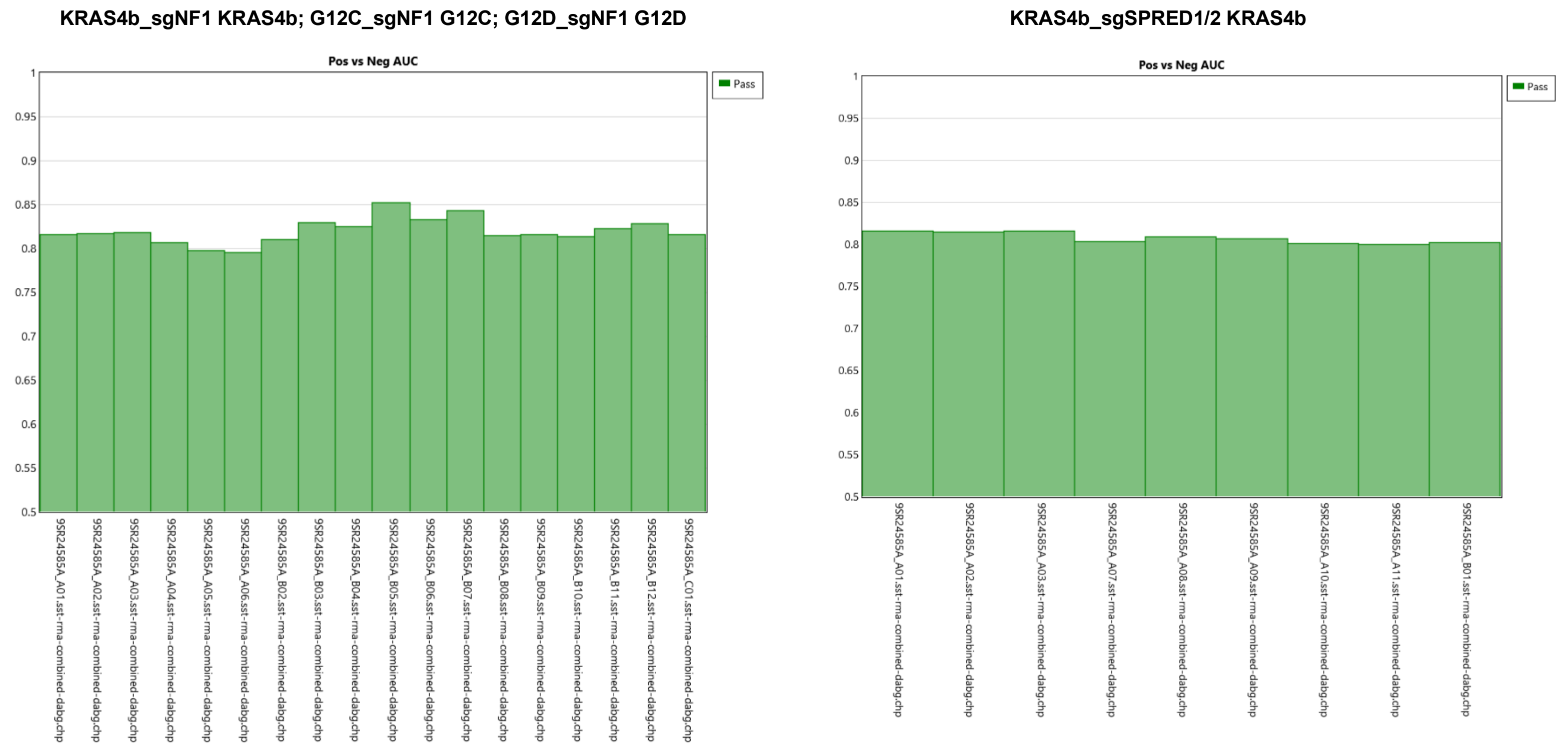

Figure S9. Quality control metrics for Clariom™ D transcriptome microarray analyses.
(A) Principal Component Analysis (PCA) on the signal (CHP) data of either the sgNF1 (left) or sgSPRED1/2 (right) knockout MEF cells and their respective parental cell lines were utilized to identify the variability in the data set. **(B)** Distribution of positive vs negative area under the curve (AUC) values for the sgNF1 (left) or sgSPRED1/2 (right) knockout MEF cells and their corresponding parental cell lines were used to determine how well the probe set summary separates the positive (exon) from the negative (intron) controls.

C.

KRAS4b_sgNF1 KRAS4b; G12C_sgNF1 G12C; G12D_sgNF1 G12D **KRAS4b_sgSPRED1/2 KRAS4b**

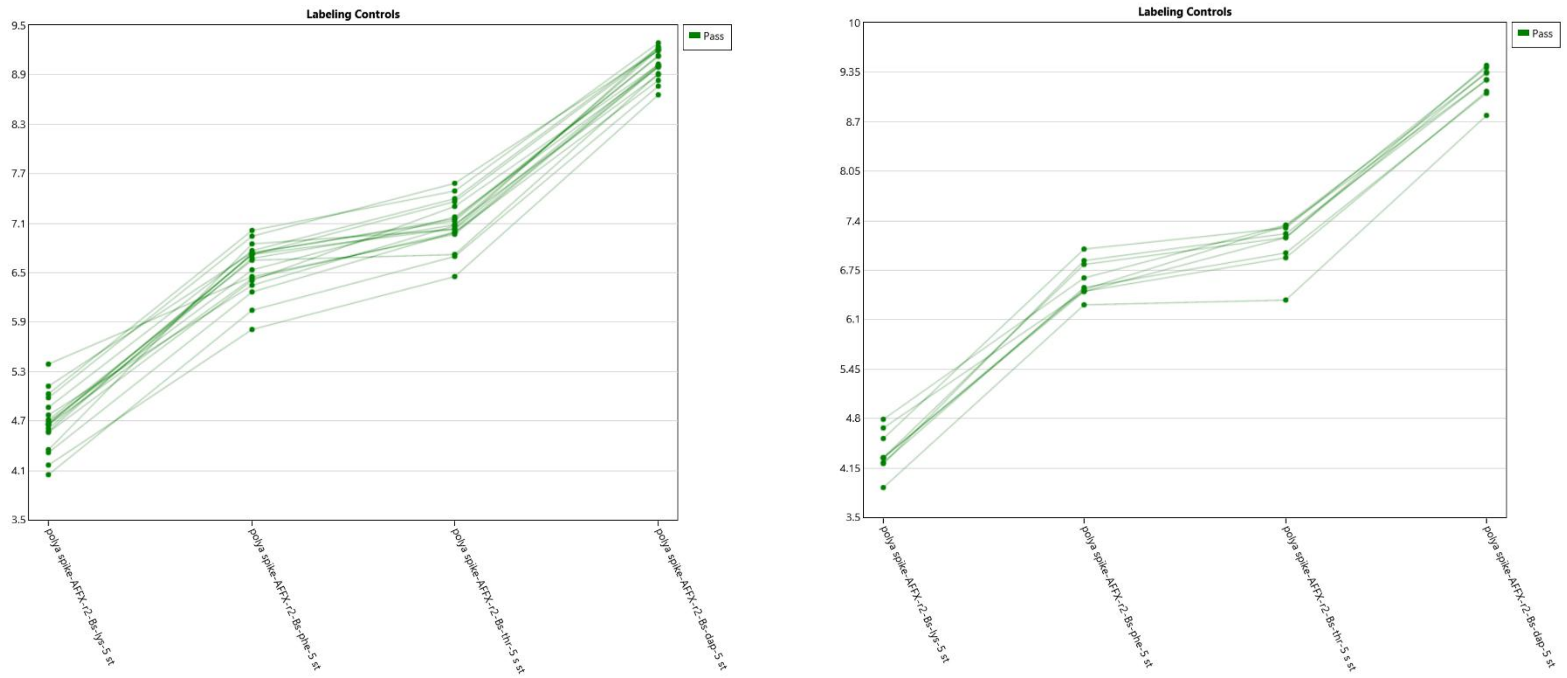

D.

KRAS4b_sgNF1 KRAS4b; G12C_sgNF1 G12C; G12D_sgNF1 G12D **KRAS4b_sgSPRED1/2 KRAS4b**

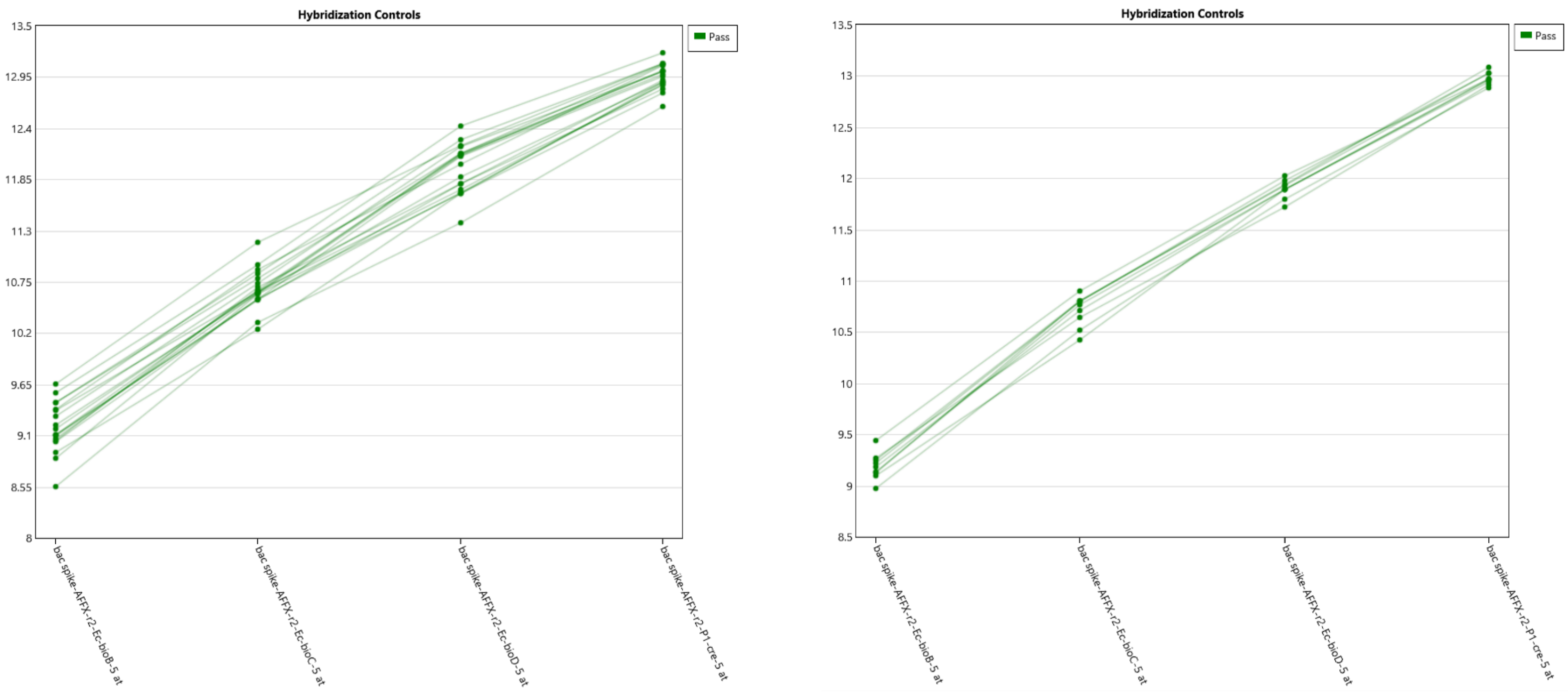

Figure S9 Cont. Quality control metrics for Clariom™ D transcriptome microarray analyses. **(C)** Poly-A RNA labeling controls of either the sgNF1 (left) or sgSPRED1/2 (right) knockout MEF cell lines and their corresponding parental cell lines were utilized to monitor the entire target labeling process. **(D)** Hybridization controls of either the sgNF1 (left) or sgSPRED1/2 (right) knockout MEF cell lines and their respective parental cell lines were used to evaluate the sample hybridization efficiency and consistency across samples.

Gene Symbol	KRAS4b vs sgNF1 KRAS4b	G12C vs sgNF1 G12C	G12D vs sgNF1 G12D	KRAS4b vs sgSRED1/2 C19	KRAS4b vs sgSRED1/2 C20
	Fold Change	Fold Change	Fold Change	Fold Change	Fold Change
Adgre5; Mir1668	-24.75	-2.53	-2.27	-9.81	-12.42
Aldh1l2	-4.82	-3.47	-3.24	-2.44	-3.23
Ar	4.73	2.04	2.62	-3.07	-3.18
Ass1	-5.75	-2.08	-2.41	-8.67	-7.28
Cdc42ep3	-5.29	-3.32	-2.52	-1.28	-1.45
Cdh11	-9.05	-23.29	-8.02	-110.03	-7.62
Col1a1	-2.56	-2.37	-2.47	-2.56	-5.32
Cpne8	3.25	2.02	2.33	1.54	1.05
Crct1	-110.04	-2.85	-2.71	1.15	-1.75
Dock8	2.51	3.85	2.34	-1.00	-1.00
Ereg	12.78	3.68	2.33	3.62	3.64
Fhl1	-5.29	-2.29	-2.60	-2.51	-3.96
Fst	-2.27	-2.56	-2.48	-1.40	-1.61
Gadd45g	-3.14	-2.09	-2.87	-2.57	-2.62
Gpr149	8.96	2.89	3.10	-1.12	1.15
Hsp25-ps1	-12.70	-10.89	-4.09	-1.03	-2.02
Hspb1	-6.96	-4.06	-2.19	1.01	-1.53
Igfbp2	-991.66	-16.18	-2.99	-15.5	-112.30
Mertk	2.53	2.60	2.27	-1.05	-1.14
Myo5b	-2.53	-2.31	-2.05	1.14	-1.23
Pcdh7	16.41	3.50	2.52	5.36	4.25
Plpp3	5.39	2.79	4.29	3.67	6.19
Ppp3ca	2.94	2.58	2.25	-1.17	-1.06
Prdm8	-2.36	-9.69	-2.08	-3.05	-3.03
Psd3	-4.74	-5.19	-5.09	-5.20	-4.85
Psme2b	-3.21	-2.86	-2.01	-2.41	-3.57
Pstpip2	-17.47	-5.34	-3.71	1.35	-2.27
Ptprk	-2.49	-6.64	-3.64	-1.40	-2.17
Rab7b	4.78	3.05	2.02	6.19	3.51
Rgmb	2.95	3.60	2.71	1.64	1.36
Rorb	2.22	4.43	12.23	-1.01	1.06
Sema3c	-81.81	-3.08	-2.16	-6.91	-66.12
Sema5a	5.49	2.32	2.58	-1.16	1.25
Serpinb9b	-8.06	-6.62	-11.15	-1.50	-2.40
α-Sma	-1.77	-4.05	-3.58	-4.21	-2.78
Slit2	8.48	4.55	2.30	-1.00	-1.00
Slurp1	-373.44	-2.83	-4.03	-13.25	-201.14
Spcs3	-2.77	-5.03	-3.24	-5.00	-6.37
Spp1	132.56	13.46	3.48	3.95	3.19
Synj2	2.08	3.08	3.28	1.14	1.31
Tagln	-4.36	-16.94	-5.01	-3.20	-2.87
Tgfb2	-12.32	-2.09	-2.23	-5.52	-12.13
Tgfb3	-3.47	-2.31	-2.61	-3.93	-4.49
Xirp2	-7.32	-11.62	-3.19	1.13	-3.27
Zfp345	3.99	2.15	2.66	1.72	3.67

Supplementary Table S1. Common genes shared amongst the sgNF1 and sgSPRED1/2 knockout MEF cell lines.

Cell Line	Cell Type	NF1	SPRED1/2	KRAS	NRAS	HRAS	BRAF
KRAS4b^{WT} MEF	MEF	WT	WT	WT	-	-	WT
KRAS^{G12C} MEF	MEF	WT	WT	G12C	-	-	WT
KRAS^{G12D} MEF	MEF	WT	WT	G12D	-	-	WT
sgNF1 KRAS4b^{WT}	MEF	DEL	WT	WT	-	-	WT
sgNF1 KRAS^{G12C}	MEF	DEL	WT	G12C	-	-	WT
sgNF1 KRAS^{G12D}	MEF	DEL	WT	G12D	-	-	WT
sgSPRED1/2 KRAS4b^{WT}	MEF	WT	DEL	WT	-	-	WT
NF1^{R1278P} KRAS4b^{WT}	MEF	R1278P	WT	WT	-	-	WT
ipnNF95.11c	Peripheral Nerve	c.1756delACTA/ Heterozygous	WT	WT	WT	WT	WT
ipNF95.bC	Plexiform Neurofibroma	c.1756delACTA/ LOH	WT	WT	WT	WT	WT
ipNF05.5	Plexiform Neurofibroma	c.3456_3457 insA/LOH	WT	WT	WT	WT	WT
sgNF1 ipnNF95.11c	Peripheral Nerve	DEL	WT	WT	WT	WT	WT
NF1^{R1276P} ipnNF95.11c	Peripheral Nerve	R1276P	WT	WT	WT	WT	WT
COLO792	Melanoma	W1236R	WT	WT	WT	WT	CNA (6)
D04	Melanoma	WT	WT	-	Q61L	-	-
M230	Melanoma	E1538X	WT	-	-	-	-
MeWo	Melanoma	Q1336*/ Hemizygous	WT	WT	WT	WT	WT
WM793	Melanoma	WT	WT	WT	WT	WT	V600E
WM3622	Melanoma	L1773fs	WT	-	-	-	-
WM3918	Melanoma	Homozygous deletion of C-term	WT	-	-	-	-

Supplementary Table S2. Mouse and human-derived cell lines and their corresponding RAS-related mutational status. MEF: mouse embryonic fibroblasts, WT: wild-type, LOH: loss of heterozygosity, CNA: copy number alterations (total copy number)

CRISPR Cell Line	Cell Type	Clone(s)	Indel Spectrum
sgNF1 KRAS4b^{WT}	MEF	5	14 base deletion<br>6 base deletion<br>5 base deletion<br>2 base deletion<br>1 base insertion
sgNF1 KRAS4b^{WT}	MEF	12	1 base insertion<br>2 base insertion w/C substitution<br>4 base deletion<br>2 base deletion<br>1 base deletion
sgNF1 KRAS4b^{WT}	MEF	16	4 base deletion<br>2 base deletion
sgNF1 KRAS4b^{WT}	MEF	27	23 base deletion<br>16 base deletion<br>5 base deletion/A base change<br>4 base deletion<br>1 base deletion/C base change
sgNF1 KRAS4b^{G12C}	MEF	30	4 base deletion<br>2 base deletion
sgNF1 KRAS4b^{G12D}	MEF	9	10 base deletion<br>4 base deletion<br>2 base deletion
sgSPRED1 KRAS4b^{WT}	MEF	7	AG replacement
sgSRPED1/2 KRAS4b^{WT}	MEF	19	4 base deletion<br>1 base insertion
sgSRPED1/2 KRAS4b^{WT}	MEF	20	1 base insertion
NF1^{R1278P} KRAS4b^{WT}	MEF	19 and 26	Homozygous for R1278P SNP
sgNF1 ipnNF95.11c	Peripheral Nerve	5	C>T base change
NF1^{R1276P} ipnNF95.11c	Peripheral Nerve	49	Homozygous for R1276P SNP

Supplementary Table S3. On-Target NGS confirmation analysis of the CRISPR edit(s) in the sgNF1 and sgSPRED1/2 knockout or the NF1 arginine finger GRD knockin mutation in the MEF and peripheral nerve cell lines.

CRISPR Name	gRNA Sequence (5' to3')	ss-Oligo Sequence
Mouse sgNF1.1	GTTGTGCTCGGTGCTGACTT	N/A
Mouse sgSPRED1.3	AATGCCTCGATCAAAAGCCC	T**OF**CCACTGGAAGATCGATGACAAGAAGTTTGGCCTTACCTTTCAGAGTCCTGCTGATGCCAGGGCTTAGGATCGAGGCATTCGAAGAGCTATAGAG**EF**T
Mouse sgSPRED2.6	AGATGCACGAGCCTTTGACA	T**ZE**GAAGGTTGATAACAGGAAGTTTGGACTTACTTTCCAAAGTCCTGCAGATGCATGAGCCTTTGACAGGGGCGTGAGAAAAGCCATTGAAGACCTT**FZ**A
Mouse NF1.1_R1278P	TCCATGCAGACTCTCTTTCG	T**FO**CAGCTGCTCTGGAACATGTTTTCTAAGGAGGTAGAATTGGCAGACTCCATGCAGACTCTCTTTCCAGGCAACAGCTTGGCCAGTAAGATAATGA**OF**T
Human sgNF1_3	CCGCGCACAGGCCGGTGGAA	C**EE**CCCACCCTTCCCTCCGCCGCCCCCCGGCCGCGGGGAGGACATGGCCGCGCACAGGCCGGTGTAATGGGTCCAGGCCGTGGTCAGCCGCTTCGAC**EF**G
Human NF1_4_R1276P	TCCATGCAGACTCTCTTCCG	C**FF**AATGAATGATACAAACCTTGAAACAGAATGTCATTATTTTACTGGCCAAGCTGTTGCCTGGGAAGAGAGTCTGCATGGAGTCTGCCAATTCTAC**ZZ**C

*Phosphorothioate modifications of the ss-Oligo donors are labeled in **blue**

Phosphorothioate Modified Nucleotides	IUPAC Symbols
Phosphorothioate-A	F
Phosphorothioate-C	O
Phosphorothioate-G	E
Phosphorothioate-T	Z

Supplementary Table S4. ***In vitro*** **transcribed (IVT) guide RNA (gRNA) and single-stranded Oligo sequences for CRISPR knockout and knockin edits.**

CRISPR Edit	Cell Line	Indel Percentage
Mouse sgNF1.1	KRAS4b^{WT}	35.6
Mouse sgNF1.1	KRAS^{G12C}	92.0
Mouse sgNF1.1	KRAS^{G12D}	93.0
Mouse sgSPRED1.3	KRAS4b^{WT}	62.3
Mouse sgSPRED2.6	KRAS4b^{WT}	72.0
Mouse NF1.1_R1278P	KRAS4b^{WT}	60.9
Human sgNF1_3	ipnNF95.11c	41.9
Human NF1_4_R1278P	ipnNF95.11c	74.1

Supplementary Table S5. On-target cleavage efficiencies of CRISPR-mediated Indel generation in the stable pool populations determined by NGS.

Antibody	Company	Catalog Number	Dilution
4E-BP1 (P-Thr37/46)	Cell Signaling Technology	2855	1:1000
4E-BP1 (P-Ser65)	Cell Signaling Technology	9456	1:1000
AKT (P-Ser473)	Cell Signaling Technology	4060	1:400
α-SMA	Cell Signaling Technology	19245	1:500
β-Actin	Sigma Aldrich	A5441	1:5000
CDH11 (Human)	Cell Signaling Technology	13577	1:250
CDH11 (Mouse)	Cell Signaling Technology	4442	1:250
COL1A1 (Human)	Cell Signaling Technology	39952	1:500
COL1A1 (Mouse)	Cell Signaling Technology	94144	1:500
ERK1/2 (P-Thr202/Tyr204)	Cell Signaling Technology	4370	1:1000
GAPDH	Cell Signaling Technology	2118	1:1000
IGFBP2	Abcam	ab188200	1:500
MEK1/2 (P-Ser217/221)	Cell Signaling Technology	9121	1:500
MRAS	Abcam	ab176570	1:500
Neurofibromin 1	Cell Signaling Technology	14623	1:200
$p70^{S6K}$ (P-Thr389)	Cell Signaling Technology	9205	1:250
PRAS40 (P-Thr246)	Cell Signaling Technology	2997	1:500
RAS (Pan)	Cytoskeleton	Part# AESA02	1:200
RASA1	Santa Cruz Biotechnology	sc-63	1:500
RASA2	Cell Signaling Technology	36342	1:500
RRAS (Human)	Cell Signaling Technology	8446	1:500
RRAS (Mouse)	Abcam	ab191791	1:500
RRAS2	Novous Biologicals	H00022800-M01	1:400
S6RP (P-Ser235/236)	Cell Signaling Technology	4858	1:500
S6RP (P-Ser240/244)	Cell Signaling Technology	5364	1:500
SPRED1	Sigma Prestige	HPA042193	1:500
TAGLN	Abcam	ab14106	1:500
Tuberin/TSC2 (P-Ser939)	Cell Signaling Technology	3615	1:400

Supplementary Table S6: Primary antibodies utilized in immunoblotting analyses.

SI REFERENCES

1. B. Garman *et al.*, Genetic and Genomic Characterization of 462 Melanoma Patient-Derived Xenografts, Tumor Biopsies, and Cell Lines. *Cell Rep* **21**, 1936-1952 (2017).
2. M. Herlyn *et al.*, Characteristics of cultured human melanocytes isolated from different stages of tumor progression. *Cancer Res* **45**, 5670-5676 (1985).
3. M. H. Nissan *et al.*, Loss of NF1 in cutaneous melanoma is associated with RAS activation and MEK dependence. *Cancer Res* **74**, 2340-2350 (2014).
4. K. Satyamoorthy *et al.*, Melanoma cell lines from different stages of progression and their biological and molecular analyses. *Melanoma Res* **7 Suppl 2**, S35-42 (1997).
5. J. N. Sondergaard *et al.*, Differential sensitivity of melanoma cell lines with BRAFV600E mutation to the specific Raf inhibitor PLX4032. *J Transl Med* **8**, 39 (2010).
6. I. Vujic *et al.*, Metformin and trametinib have synergistic effects on cell viability and tumor growth in NRAS mutant cancer. *Oncotarget* **6**, 969-978 (2015).
7. J. M. Silva, C. Bulman, M. McMahon, BRAFV600E cooperates with PI3K signaling, independent of AKT, to regulate melanoma cell proliferation. *Mol Cancer Res* **12**, 447-463 (2014).
8. J. M. Silva, M. McMahon, The fastest Western in town: a contemporary twist on the classic Western blot analysis. *J Vis Exp* 10.3791/51149, e51149 (2014).
